# High-Fat Diet and Altered Radiation Response

**DOI:** 10.3390/biology14040324

**Published:** 2025-03-22

**Authors:** Jiraporn Kantapan, Takanori Katsube, Bing Wang

**Affiliations:** 1 Molecular Imaging and Therapy Research Unit, Department of Radiologic Technology, Faculty of Associated Medical Sciences, Chiang Mai University, Chiang Mai 50200, Thailand; 2 Institute for Radiological Science, National Institutes for Quantum Science and Technology (QST), Chiba 263-8555, Japan; katsube.takanori@qst.go.jp

**Keywords:** radiosensitivity/susceptibility, high-fat diets, reactive oxygen species (ROS), inflammation, DNA damage, radiation

## Abstract

High-fat diets (HFDs) have become increasingly prevalent in modern societies, driving rising rates of obesity and metabolic syndrome. Concurrently, radiation exposure from medical treatments and environmental sources poses health risks shaped by both biological and environmental factors. This review explores the intersection between HFDs and radiation sensitivity/susceptibility, focusing on how diet-induced metabolic alterations influence the body’s response to radiation. Evidence from preclinical and clinical studies indicates that HFDs significantly alter metabolism, leading to increased oxidative stress and immune system dysregulation. These metabolic changes can exacerbate radiation-induced oxidative stress, inflammation, and DNA damage, potentially increasing radiation sensitivity in normal tissues. Conversely, obesity and HFD-induced metabolic disruptions may activate cellular pathways involved in DNA repair, cell survival, and inflammatory responses, fostering tumor resistance and modifying the tumor microenvironment, which may impair the efficacy of radiation therapy in cancer treatment. Understanding the interplay between diet and radiation exposure is critical for optimizing public health guidelines and improving therapeutic outcomes. These findings underscore the need for further research into dietary interventions that may mitigate radiation-associated risks.

## Simple Summary

High-fat diets (HFDs) are becoming increasingly prevalent and are a major contributor to the rising incidence of obesity and metabolic disorders, including diabetes and heart disease. At the same time, exposure to ionizing radiation (IR) from medical procedures, environmental sources, and occupational settings remains a significant public concern. This review explores the interplay between HFDs and IR, focusing on their combined effects on metabolism, immune function, and DNA repair mechanisms. We found that HFDs can exacerbate radiation-induced damage by increasing oxidative stress, inflammation, and DNA damage, potentially elevating the risk of chronic diseases. Surprisingly, in some cases, HFDs also trigger cellular changes that reduce the efficacy of radiation therapy in cancer treatment. Furthermore, maternal HFD exposure can increase radiation sensitivity in offspring, particularly males, by disrupting immune and metabolic functions. Understanding the influence of diet on radiation response is crucial for developing effective public health strategies. The timing and composition of dietary intake in the context of radiation exposure are key considerations for optimizing public health interventions, improving cancer treatment outcomes, and minimizing radiation-related health risks. We encourage collaborative dietary and lifestyle interventions aimed at reducing chronic disease risk and enhancing health outcomes, particularly among vulnerable populations. This review provides evidence to guide such efforts toward improved health and therapeutic outcomes.

## 1. Introduction

Populations receive regular exposure to radiation from medical procedures, occupational exposure, and environmental sources. Radiation sensitivity is the biological reaction of the tissues and cells to ionizing radiation (IR) in accordance with the energy of the radiation, which can harm the DNA, proteins, lipids, and some other resources inside a cell. This sensitivity differs significantly between individuals, tissues, and pathological conditions and is determined by genetic, metabolic, and environmental factors [1]. Determining radiation sensitivity minimizes damage and maximizes therapeutic and protective responses.

Healthy tissues generally exhibit resilience to low and moderate doses of radiation due to robust DNA repair mechanisms and antioxidant defense systems [2]. Efficient DNA damage response (DDR) pathways, such as homologous recombination (HR) and non-homologous end-joining (NHEJ), are essential for repairing radiation-induced double-strand breaks. At the same time, antioxidant systems such as glutathione and superoxide dismutase neutralize reactive oxygen species (ROS) generated during radiation exposure. However, radiosensitivity varies by tissue type. Rapidly dividing tissues, such as bone marrow and intestinal epithelium, are inherently more sensitive to radiation due to their high proliferative rates. However, muscle and nerve tissue are relatively resistant, owing to their lower cellular turnover [3]. Age, sex, and underlying health conditions further modulate the sensitivity to radiation. Aging, for example, decreases DNA repair and antioxidant capacity, and the presence of some senescent cells creates a pro-inflammatory environment that aggravates radiation damage [4]. Radiation susceptibility is often heightened in pathological conditions due to impaired DNA repair mechanisms, chronic inflammation, or metabolic dysregulation. Genetic disorders such as ataxia-telangiectasia (AT) and Nijmegen breakage syndrome result in extreme radiosensitivity due to defects in key DDR proteins such as ataxia-telangiectasia-mutated kinase (ATM) and nibrin (NBN) [5]. Similarly, metabolic disorders, including obesity and diabetes, exacerbate radiation sensitivity through systemic inflammation and oxidative stress. Elevated cytokines, such as TNF-α and IL-6, impair tissue recovery, while obesity-associated dyslipidemia amplifies ROS-induced cellular damage [6]. In cancer patients, radiation sensitivity presents a dual challenge: while cancer cells are often radiosensitive due to rapid proliferation and genomic instability, hypoxic regions within tumors are resistant to radiation. This resistance arises because oxygen enhances ROS-mediated DNA damage, a phenomenon known as the oxygen enhancement ratio (OER) [7]. Mutations in genes such as tumor protein p53 (TP53) and breast cancer genes 1/2 (BRCA1/2) further impair DDR pathways, increasing susceptibility to radiation-induced apoptosis [8].

In the past decades, the prevalence of high-fat diets (HFDs) has been remarkably increasing globally, and this dietary pattern is strongly associated with the risk of chronic diseases, including cardiovascular diseases, diabetes, and cancers [9,10]. HFDs, as referred to in this review, are diets characterized by a high intake of unhealthy fats, such as saturated fats (abundant in animal products and fatty meats) and trans fats (prevalent in processed foods). In contrast, this definition does not include diets that emphasize healthy fats from sources such as olive oil, avocados, nuts, and fatty fish. Notably, the emphasis here is on the health implications of these diets, their metabolic and cellular effects, rather than their caloric content alone. Obesity, a multifaceted chronic disease often driven by HFDs, is characterized by excessive body fat tissue mass and is associated with disturbances in lipid and glucose metabolism, chronic inflammation, and oxidative stress. These metabolic disruptions increase the risk of several severe diseases and reduce life expectancy. Moreover, obesity has been linked to the accumulation of DNA damage, which may contribute to the development of obesity-related conditions [11].

As HFDs become more common, the possible effects on radiation response warrant close investigation. However, there is emerging evidence for the interaction of HFD-induced metabolic changes and radiation sensitivity. Such diets affect critical cellular and systemic pathways, such as DDR, inflammation, and oxidative stress, which are major radiation response determinants. That said, biological studies of the link between HFD and radiation response have been conflicted, as both increased sensitivity and increased resistance have been observed depending on the experimental context [12,13,14,15]. This duality highlights the importance of a holistic understanding of the effect of HFDs on radiation sensitivity in both normal and malignant tissue. Such insights are critical for developing effective therapeutic strategies and assessing risks in radiation-exposed population groups via medical therapies, occupational exposures, or environmental sources. The consequences of HFDs are highly context-dependent, determined by genetic susceptibility, other environmental factors, and the amount of exposure to IR. For instance, animal-based HFDs often contain elevated cholesterol levels, which can exacerbate adverse health outcomes. This review also explores how cholesterol-enriched diets modulate the effects of IR.

To provide a comprehensive and critical analysis of the interplay between HFDs and radiation response, we conducted an extensive literature search across PubMed, Scopus, and Web of Science. The search employed a combination of keywords, including “high-fat diet”, “obesity”, “radiation response”, “DNA damage response (DDR)”, “metabolic dysregulation”, and “cancer metabolism”. We prioritized peer-reviewed articles published between 2004 and 2024, ensuring the inclusion of recent scientific advancements while incorporating seminal studies that shaped the current understanding of this field. For radiation-related studies, we performed a targeted PubMed search using the keywords “radiation”, “high-fat diet”, and “response”, which yielded 261 articles. Abstracts were systematically reviewed, and 41 representative papers were selected based on their focus on radiation sensitivity, DDR impairment, and metabolic alterations in HFD models. To clarify the concept and metabolic implications of HFDs, we conducted a separate PubMed search using the keywords “high-fat diet”, “concept”, and “review”, restricting results to publications from 2010 to 2024. This search retrieved 103 articles, from which we cited three key papers that provided conceptual frameworks and definitions of HFDs. Our selection included meta-analyses, systematic reviews, and experimental studies that offered mechanistic insights into the effects of HFD-induced metabolic dysregulation on radiation sensitivity. Additionally, we explored studies examining maternal HFD exposure and its potential impact on the radiosensitivity of offspring, a hypothesis supported by emerging research in developmental and transgenerational biology. These criteria ensured a rigorous and evidence-based evaluation of the complex interactions between HFDs, obesity-related inflammation, and radiation response.

We critically review the existing evidence for the association between HFD and radiation response, elucidate potential mechanisms, and consider the clinical implications of these findings. This review highlights how HFDs contribute to radiological outcomes by impacting metabolic, molecular, and cellular processes. Furthermore, we discuss potential strategies for mitigating adverse effects and optimizing therapeutic efficacy in the context of the rising prevalence of HFDs.

## 2. Radiation Sensitivity

Radiation sensitivity describes the extent to which cells, tissues, or organisms respond to IR and is determined by the degree of biological damage induced by a specific dose. This damage can manifest as DNA breaks, cell death, or functional impairments, with higher radiation sensitivity indicating that severe effects occur at lower doses of IR. For instance, IR primarily induces both cancer and tissue toxicity through the induction of simple and complex DNA damage in individual cells. Accurate repair, misrepair, or failure to repair this DNA damage determines the fate of each cell. Fixing DNA mutations increases the risk of malignant transformation, while irreparable DNA damage is typically lethal [16].

While radiosensitivity refers to the cellular and tissue-level response to IR, the susceptibility to radiation is a general term reflecting a person’s tendency to be affected by the harmful effects of radiation exposure due to the underlying genetic, molecular, and physiological characteristics. Significant contributors to susceptibility were mutations in DNA repair genes such as TP53, BRCA1/2, and ATM. These mutations impair the resolution of radiation-induced damage, increasing radiosensitivity and the likelihood of long-term complications such as carcinogenesis. For example, germline variants in TP53 are associated with Li-Fraumeni syndrome, which increases the risk of various cancers, including breast cancer. Similarly, mutations in BRCA1 and BRCA2 are linked to hereditary breast and ovarian cancers, and carriers of pathogenic variants in ATM have an elevated risk of developing breast cancer [17,18]. Epigenetic regulation, including DNA methylation and chromatin remodeling, is a crucial component of gene expression regulation, and hence, the expression of genes relevant to DNA repair pathways. IR may alter DNA repair genes’ expression and potentially exacerbate radiation-induced damage. For example, radiation exposure has been demonstrated to produce site-specific increases and decreases in DNA methylation, leading to increases and decreases in gene expression [19]. Genetic disorders such as ataxia-telangiectasia (AT) and Nijmegen breakage syndrome (NBS) are characterized by deficiencies in DNA repair pathways, leading to heightened sensitivity to IR. Individuals with these conditions exhibit increased radiation sensitivity due to impaired DNA damage response mechanisms [5]. Furthermore, decreased cellular antioxidants (such as glutathione, superoxide dismutase, and catalase) can further worsen oxidative damage due to insufficient neutralization of ROS produced upon radiation exposure. Such imbalance leads to increased cellular damage and enhanced sensitivity to radiation [20]. Concerning susceptibility to IR, epigenetic changes and epigenetic mechanisms may also play a role, as well as genetic predispositions to DNA damage repair potential and deficiencies in antioxidant capacity.

The distinction between radiation sensitivity and susceptibility remains a topic of discussion. Some authors suggest that tissue reactions related to cell death (due to radiation exposure) should be described as sensitivity to radiation. In contrast, an individual’s genetic predisposition to subsequent long-term tissue effects, such as radiation-induced cancers, should be referred to as susceptibility [21,22]. However, despite this distinction, these terms are frequently used interchangeably in the scientific literature. For this review, radiosensitivity encompasses both the biological reaction to IR, including cell death and tissue damage, and an individual’s risk for radiation-induced cancer.

### Mechanism of Radiosensitivity

Radiosensitivity is a complex of molecular, cellular, and physiological mechanisms by which IR affects cell response. This response is dictated mainly by the efficacy of detecting, repairing, or tolerating radiation-induced damage. The key mechanisms that govern radiosensitivity include the following:

1. DNA Damage and Repair:
  DNA damage, particularly double-strand breaks (DSBs), is among the most lethal and mutagenic forms of damage at the core of radiosensitivity. If DSBs are left unrepaired or are improperly repaired, they can lead to genomic instability, apoptosis, or even oncogenesis. The DNA repair system mitigates this damage through two primary pathways:
  - NHEJ: An error-prone process active throughout the cell cycle.
  - HR: A high-fidelity mechanism restricted to the S and G2 phases.
  Mutations in DNA repair genes, including BRCA1, BRCA2, and ATM, contribute significantly to diminished repair capacity, dramatically increasing radiosensitivity. For example, some genes, such as BRCA1 and BRCA2, play key roles in homologous recombination and DNA repair; mutations in them can increase cancer risk [23]. Similarly, defects in ATM, a pivotal regulator of the DNA damage response, have a profound impact on increasing sensitivity to IR, highlighting the severity of the issue [24].
2. ROS and Oxidative Stress:
  IR also generates ROS, which damage cellular macromolecules, including DNA, lipids, and proteins, leading to oxidative stress. While antioxidant systems such as glutathione, superoxide dismutase, and catalase work to mitigate ROS, an imbalance favoring oxidative stress exacerbates radiosensitivity, underlining its importance in radiation biology [25].
3. Epigenetic Modifications:
  Epigenetics such as DNA methylation modifiers also played a vital role in determining radiosensitivity. These modifications modulate gene expression and impact critical processes such as DNA repair, apoptosis, and oxidative stress handling. Dysregulation of these epigenetic effectors can amplify radiation damage and contribute to cancer progression [19].
4. Immune System Modulation:
  The immune system further influences radiosensitivity. IR can modulate the immune microenvironment by recruiting immune cells, such as macrophages and T-cells, that either stimulate tumor growth or mount an anti-tumor response. Chronic IR exposure can lead to sustained inflammation, resulting in tissue damage and creating a microenvironment conducive to carcinogenesis [26].
5. Radiation Adaptation:
  Cells exposed to sublethal IR may develop in acquiring a condition termed radiation adaptation, which promotes resistance to a second exposure to IR. This adaptive response is achieved by upregulating DNA repair genes, antioxidant defenses, or other protective mechanisms. Understanding and leveraging this phenomenon could provide opportunities to optimize radiotherapy, minimizing damage to healthy tissues while maintaining therapeutic efficacy [27].
6. Intrinsic and Extrinsic Modulating Factors:
  Several intrinsic and extrinsic factors also modulate radiosensitivity. The cell cycle phase plays a critical role, with cells in the G2/M phase being the most radiosensitive due to reduced DNA repair capacity, while cells in the late S phase exhibit greater resistance. Oxygen availability is another major determinant; high oxygen levels amplify the generation of ROS, increasing DNA damage, whereas hypoxia promotes radioresistance by reducing oxidative injury. Furthermore, cell type significantly affects radiosensitivity, with rapidly proliferating cells, such as those in the gastrointestinal tract or bone marrow, being more sensitive due to their limited ability to repair DNA damage before division [28].

7. Physiological and Lifestyle Factors:
  Physiological and lifestyle factors can significantly impact one’s radiosensitivity, including age, diet, smoking habits, preexisting illnesses, and health status [29]. For instance, older people or people with underlying conditions such as diabetes may demonstrate increased sensitivity to radiation because of impaired DNA repair systems and decreased antioxidant systems. Diet, particularly HFDs, has also been shown to modulate radiosensitivity. HFDs are associated with increased oxidative stress, chronic inflammation, and metabolic imbalances, all of which can exacerbate radiation-induced damage. Excessive dietary fats can disrupt cellular homeostasis by enhancing ROS production and impair normal DNA repair processes, further sensitizing cells to IR. Most importantly, individual patient factors, including these lifestyle factors, are estimated to explain 81–90% of the variance in tissue damage in breast cancer patients during radiotherapy and thus provide a significant barometer of the need for personalization of treatment accounting for these variables [30].

In summary, radiosensitivity represents a complex and non-linear inter-functional phenomenon conditioned by genetic, epigenetic, molecular, and physiological mechanisms. Knowledge of these processes helps define avenues for better use of radiation therapy, to limit damage to healthy tissue, and to introduce targeted approaches to ameliorate radiation-induced lesions.

## 3. Impact of HFD on Cellular Function

### 3.1. Effect of HFDs on Metabolic Dysregulation and Inflammation

The global prevalence of obesity and metabolic and chronic diseases is closely linked to the over-consumption of HFDs. The adverse impact of HFDs on metabolic and cellular functions has been a suggested and studied causal factor of type 2 diabetes (T2DM). Severe dietary fat intake, in particular, saturated fats, sets off a chain of metabolic perturbations resulting in obesity and the development of insulin resistance, an early step in the pathogenesis of type 2 diabetes [9,31]. Ectopic fat accumulation in tissues such as the liver, muscle, and pancreas adds further metabolic insult, with impaired glucose homeostasis leading to progressively reduced insulin sensitivity [32]. These highlight the need for early intervention and preventative measures to halt the self-perpetuating cycle of progressive metabolic dysregulation.

One defining feature of HFD-induced metabolic dysfunction is the increased presence of free fatty acids (FFAs) in circulation. These FFAs drive triglyceride (TG) accumulation in key metabolic tissues such as the adipose tissue, liver, pancreas, and skeletal muscle. Excess FFAs are re-esterified in adipose tissue into triglycerides, promoting fat storage and contributing to obesity. As obesity develops, adipose tissue expands through two processes: hypertrophy (enlargement of existing fat cells) and hyperplasia (increased number of adipocytes) [33]. This excess fat storage is often caused by a nutrient supply exceeding the body’s energy requirements. Obesity, a global health challenge, is associated with systemic low-grade inflammation and insulin resistance. Several mechanisms are involved in the progression of obesity. As adipose tissue expands during weight gain, enlarged adipocytes experience hypoxia, increasing cell death [34]. This process triggers the release of FFAs, ROS, and pro-inflammatory cytokines from adipocytes and the surrounding adipose tissue. Research shows that macrophage infiltration significantly increases in obese adipose tissue, with macrophages constituting over 50% of the total cell population in cases of severe obesity [35]. This substantial presence highlights the pivotal role of macrophages in driving and sustaining inflammation within adipose tissue.

Obese adipose tissue serves as a significant source of pro-inflammatory adipokines, including monocyte chemotactic protein-1 (MCP-1), tumor necrosis factor-alpha (TNF-α), interleukin-1 beta (IL-1β), and interleukin-6 (IL-6) [36]. The mechanism underlying this inflammatory response involves FFAs activating Toll-like receptor 4 (TLR4) expression on resident adipocytes and macrophages. This activation triggers downstream signaling cascades, such as the myeloid differentiation primary response gene 88 (MyD88) and TIR domain-containing adapter-inducing interferon-β (TRIF) pathways, which amplify endoplasmic reticulum (ER) stress, ROS production, and pro-inflammatory cytokine secretion. Furthermore, FFAs stimulate nuclear factor-kappa B (NF-κB) and p38 mitogen-activated protein kinase (MAPK) signaling pathways, exacerbating oxidative stress and inflammatory cytokine production [37,38]. MCP-1, a key adipokine, attracts monocytes to inflamed adipose tissue by binding to the C-C motif chemokine receptor 2 (CCR2). Once recruited, these monocytes differentiate into macrophages at the site of inflammation. In obesity, adipose tissue macrophages undergo a phenotypic shift from the anti-inflammatory M2 state to the pro-inflammatory M1 state. M1 macrophages release additional pro-inflammatory cytokines, including MCP-1, IL-1β, and IL-6, creating a self-perpetuating cycle of inflammation that attracts more monocytes as adipocyte size increases and inflammatory conditions worsen [37,38]. Macrophages recruited to clear cellular debris from dying adipocytes form crown-like structures (CLS) around these cells. This process impairs macrophage regulatory functions as they absorb lipids from the dead adipocytes and exacerbate inflammation and dysfunction within the adipose tissue [36]. The prevalence of CLS strongly correlates with systemic metabolic disturbances and chronic inflammation [39]. Chronic overnutrition also causes an alteration in adipose tissue function with an increase in circulating FFAs, ROS, and pro-inflammatory cytokines. These changes create a systemic environment of low-grade chronic inflammation involved in the pathogenesis of diseases such as type 2 diabetes and cardiovascular disease. This inflammatory state underscores the pivotal role of dysfunctional adipose tissue in the progression of obesity-related diseases.

Insulin, produced by pancreatic β-cells, is critical in maintaining glucose and lipid homeostasis by responding to circulating glucose and fatty acid levels. After a meal, increased blood glucose stimulates insulin release from β-cells. Insulin binds to receptors on skeletal muscle, adipose tissue, and liver cells, promoting glucose uptake for energy metabolism, fatty acid synthesis, and protein production [40]. In obesity, chronic inflammation disrupts insulin signaling due to elevated levels of pro-inflammatory cytokines. TNF-α is critical in disrupting insulin signaling by targeting key components of the insulin signaling cascade. One of its primary actions is the activation of signaling pathways, including IKK, p38 MAPK, JNK, and PKC. These pathways promote the phosphorylation of serine residues on insulin receptor substrate (IRS) proteins, a modification that interferes with normal tyrosine phosphorylation. This change prevents IRS proteins from effectively transferring insulin signals, inhibiting insulin signaling in key metabolic tissues, including the skeletal muscle, adipose tissue, and the liver [36]. Moreover, TNF-α increases protein tyrosine phosphatase 1B (PTP1B) expression, an enzyme that dephosphorylates the tyrosine residues on insulin receptors. This enzyme activity also represses the function of the insulin receptor by decreasing its phosphorylation state and thus further prevents the output of the insulin signaling pathway [41]. Interestingly, serine phosphorylation of IRS proteins and tyrosine dephosphorylation of the insulin receptors heavily inhibit glucose uptake and metabolism, constituting insulin resistance. Similarly, increased IL-6 in obesity contributes considerably to insulin resistance by activating the JAK-STAT signaling pathway. This activation induces the expression of suppressors of cytokine signaling proteins (SOCS), SOCS1 and SOCS3, which interfere with the interaction between insulin receptors and IRS proteins, impairing the downstream signaling necessary for effective insulin action [42]. SOCS proteins can also disrupt insulin receptor kinase activity, compromising glucose metabolism and cellular energy balance. IL-1β, another pro-inflammatory cytokine elevated in obesity, further contributes to the impairment of insulin signaling. IL-1β activates p38 MAPK, a kinase that phosphorylates serine residues on IRS1/2 proteins. This serine phosphorylation disrupts normal tyrosine phosphorylation, a key step in insulin signal transduction, leading to reduced insulin sensitivity [43].

Insulin resistance, a condition where cells fail to respond to insulin effectively, has significant health implications. Although TNF-α may impair β-cell insulin sensitivity through nitric oxide pathways, evidence of inflammation directly causing β-cell dysfunction remains inconclusive [44]. However, these inflammatory pathways simultaneously impair insulin signaling, which promotes insulin resistance. These inhibit glucose uptake, leading to increased blood glucose levels and compensated for by β-cells overproducing insulin, eventually leading to exhaustion and dysfunction of β-cells. This pathway leads to chronic elevation of blood glucose, a hallmark of insulin resistance and metabolic syndrome [45]. Research shows that insulin resistance, reduced insulin sensitivity, and elevated blood glucose levels can precede the diagnosis of T2DM by up to 13 years [46]. Insulin resistance is further linked to increased risks of heart failure, tumor progression, and cognitive decline, elevating the likelihood of Alzheimer’s disease [47]. These health issues underscore the importance of understanding and addressing insulin resistance.

In addition, obesity-induced excess FFAs are often taken up by the liver, where they are stored as fat droplets, contributing to the development of non-alcoholic fatty liver disease (NAFLD). This condition is characterized by hepatic steatosis, where lipid accumulation within hepatocytes exceeds 5% of liver weight, impairing liver function and increasing the risk of fibrosis and cirrhosis [48]. HFDs exacerbate this process by stimulating hepatic de novo lipogenesis, wherein carbohydrates and excess FFAs are converted into triglycerides. This metabolic shift increases the production and secretion of very low-density lipoproteins (VLDL), elevating serum FFA, triglyceride, and cholesterol levels [49]. The excess FFAs and lipid intermediates contribute to fat deposition and promote lipotoxicity, oxidative stress, and mitochondrial dysfunction in hepatocytes. These conditions activate inflammatory pathways, including NF-κB and JNK signaling, which exacerbate liver inflammation and insulin resistance [50]. Over time, the sustained lipid overload impairs hepatic insulin signaling, further disrupting glucose and lipid homeostasis. Additionally, cholesterol accumulation within the liver can exacerbate liver injury by inducing the formation of cholesterol crystals, which activate Kupffer cells and hepatic stellate cells, contributing to fibrosis development [51]. This cumulative metabolic burden links NAFLD with systemic metabolic disorders such as type 2 diabetes and cardiovascular disease, highlighting the profound impact of obesity and HFDs on liver health and overall metabolic function.

### 3.2. Effect of HFDs on Oxidative Stress

HFDs are closely associated with increased oxidative stress, characterized by an imbalance between the excessive production of ROS and the body’s antioxidant defenses [52]. Preclinical research revealed that adipose tissue from HFD-fed mice exhibited significantly higher ROS levels than controls. This oxidative stress was linked to increased macrophage infiltration and secretion of pro-inflammatory cytokines, exacerbating systemic inflammation and promoting metabolic dysfunction [53]. Similarly, human studies found that individuals consuming diets high in saturated fats displayed elevated oxidative stress markers, including malondialdehyde (MDA) and oxidized low-density lipoprotein (LDL). These markers were strongly associated with an increased risk of cardiovascular diseases [54]. Accumulating evidence has shown that HFDs disrupt metabolic homeostasis, especially when accompanied by a high ratio of fatty acids that are mostly saturated fats. Such disturbance causes the overproduction of ROS to the extent that antioxidant enzymes can no longer stabilize the system [55,56]. Oxidative damage by ROS targets vital cellular entities such as lipids, proteins, and DNA. This damage causes lipid peroxidation and leads to cell membrane damage, protein oxidative modifications that induce a potential loss of enzymatic activity, and possible disruption of signaling pathways. One of the foremost consequences of HFDs, oxidative stress, is a significant inducer of cellular apoptosis and activates genomic instability. These can result in chronic diseases, even cancer, highlighting the potential severity of our research on human health. The oxidative stress induced by HFDs activates several protective and inflammatory signaling pathways, including those involving heat shock proteins (HSPs) and MAPKs. Although these pathways are protective initially, persistent activation leads to chronic inflammation, additional ROS production, and metabolic disruption [57,58]. Furthermore, studies have shown that ROS-mediated oxidative stress induced by HFDs also accounts for impaired mitochondrial function, leading to decreased ATP production and mitochondrial dysfunction [59]. As a result, energy imbalances, as well as systemic metabolic disorders, arise.

Mitochondria are essential for ATP production, energy balance, and ROS management, playing a central role in cellular metabolism. They adapt dynamically to metabolic demands through mitophagy, apoptosis, fusion, and fission [60]. However, excessive energy substrates, such as those provided by HFDs, can overwhelm this balance, leading to mitochondrial dysfunction that disrupts lipid and glucose metabolism. Adipocytes rely on normal mitochondrial function to maintain energy storage and expenditure equilibrium, but HFD-induced mitochondrial impairments hinder this capacity, contributing to systemic metabolic dysregulation. Excessive dietary fats disrupt mitochondrial dynamics by increasing ROS production and impairing energy production [61]. Dysregulation in mitochondrial fusion and fission can further exacerbate dysfunction. While fusion promotes organelle elongation and interconnectivity, excessive fusion leads to dysfunctional elongation, reducing functionality. Fission, in contrast, splits mitochondria into smaller fragments, mitigating abnormality, but at the cost of reduced energy generation [62]. Prolonged dysfunction can damage mitochondrial DNA, amplify ROS production, and trigger apoptosis.

Mitochondria in obese individuals exhibit significant functional and structural differences compared to those in lean individuals. Obesity impairs mitochondrial biogenesis, increases lipid peroxidation, and generates metabolites, such as diacylglycerol (DAG), ceramides, and acetyl-CoA, due to incomplete fatty acid oxidation [63]. Mitochondrial dysfunction is most pronounced in key metabolic tissues, including adipose tissue, skeletal muscle, and liver. In obese adipose tissue, mitochondrial dynamics are disrupted by reduced expression of fusion proteins such as mitofusin 1 (Mfn1) and mitofusin 2 (Mfn2), coupled with increased levels of the fission protein dynamin-related protein 1 (Drp1). This imbalance alludes to an overactive mitochondrial fission, which leads to segmented mitochondria with a diminished ability to generate ATP [64]. In addition, fragmentation leads to increased production of ROS, promoting oxidative stress and the release of pro-inflammatory cytokines, thus worsening systemic inflammation. In skeletal muscle, increased circulating FFAs characteristic of obesity induce a compensatory force to regulate lipid overload by increasing the rate of β-oxidation [65]. Nevertheless, this reaction stresses mitochondria beyond their limits, reducing their mass, respiratory capacity, and effectiveness in ATP production. These deficits impair glucose uptake and use and promote insulin resistance. Upregulated Drp1 increases mitochondrial fission in the liver, disrupting energy production and respiratory efficiency. These impair lipid metabolism, causing the accumulation of triglycerides and FFAs, which have become known hallmarks of NAFLD [66]. Savini et al. (2013) investigated the effects of HFDs on hepatic oxidative stress [67]. Mice administered an HFD displayed significantly increased lipid peroxidation markers, mitochondrial dysfunction hallmarks, and diminished antioxidant enzyme levels. This caused oxidative damage, which led to NAFLD [68]. Excessive mitochondrial fragmentation also disrupts calcium homeostasis by promoting calcium transfer from the endoplasmic reticulum (ER) to mitochondria via mitochondria-associated ER membranes (MAMs). Calcium overload worsens mitochondrial dysfunction, triggers ER stress, and induces oxidative stress and apoptosis. These interconnected processes establish a vicious cycle, exacerbating hepatic steatosis and driving progression to more severe conditions, such as non-alcoholic steatohepatitis (NASH) and fibrosis [69].

HFDs significantly elevate oxidative stress through their impact on mitochondrial metabolism and FFA influx. The excessive FFAs supplied to mitochondria by HFDs are metabolized via β-oxidation, generating large quantities of acetyl-CoA [70]. This acetyl-CoA enters the tricarboxylic acid (TCA) cycle, oxidizing it to produce reducing equivalents such as NADH and FADH_2_. These reducing equivalents act as electron donors for the mitochondrial electron transport chain (ETC), driving ATP synthesis under normal metabolic conditions. However, the excessive input of reducing equivalents in the context of HFDs overwhelms the ETC’s capacity to transfer electrons efficiently. When the ETC becomes overloaded, electrons “leak” prematurely from the electron transport complexes (primarily complexes I and III), reacting with molecular oxygen to generate superoxide (O_2_^−^), a primary form of ROS [71]. The overproduction of ROS disrupts cellular homeostasis by damaging lipids, proteins, and DNA. This imbalance between ROS generation and antioxidant defenses creates a pro-oxidative state, impairing mitochondrial function and contributing to metabolic dysfunction. A study demonstrated that HFD-fed rats exhibited reduced activity of key antioxidant enzymes, such as superoxide dismutase and catalase, in the liver. These reductions correlated with elevated oxidative stress markers, including MDA, a byproduct of lipid peroxidation [72]. Conversely, research showed that antioxidant supplementation with N-acetylcysteine (NAC) in HFD-fed mice effectively reduced ROS levels, improved mitochondrial function, and alleviated insulin resistance [73]. These findings underscore the critical role of oxidative stress in HFD-induced metabolic disorders and highlight the therapeutic potential of antioxidant strategies to mitigate its effects. In addition to ROS, HFDs activate inducible nitric oxide synthase (iNOS), leading to the overproduction of nitric oxide (NO) and the formation of reactive nitrogen species (RNS), compounding oxidative stress [74]. Elevated ROS levels also stimulate pro-inflammatory pathways, including NF-κB activation, which increases the expression of pro-inflammatory mediators such as TNF-α, IFN-γ, and iNOS. This cascade creates a feedback loop of oxidative stress and inflammation, contributing to metabolic dysfunction and the pathogenesis of obesity-related diseases [75].

In summary, the mechanism by which HFDs cause oxidative stress involves mitochondrial dysfunction, ER stress, activation of NADPH oxidase, and diminished antioxidant defenses. These drive metabolic and cellular dysfunctions such as insulin resistance, fatty liver, and systemic inflammation, which underlines the direct connection between dietary fats, oxidative stress, and chronic diseases.

### 3.3. The Impact HFDs on Key Metabolic Regulators

HFDs significantly disrupt metabolic homeostasis by altering lipid metabolism, energy balance, and key signaling pathways. The dysregulation of AMP-activated protein kinase (AMPK), sterol regulatory element-binding protein-1c (SREBP-1c), and peroxisome proliferator-activated receptor gamma (PPARγ) plays a central role in metabolic dysfunction and disease progression, including obesity, insulin resistance, and cancer. These metabolic regulators control lipid storage, fatty acid oxidation, and glucose utilization, and their modulation by HFDs contributes to systemic metabolic disorders.

1. AMPK: Master Regulator of Cellular Energy Balance and Lipid Metabolism

AMPK is a crucial energy sensor that regulates ATP homeostasis by coordinating anabolic and catabolic pathways. Activated under low energy conditions (e.g., fasting, exercise), AMPK promotes catabolic processes while inhibiting anabolic pathways. Specifically, AMPK phosphorylates acetyl-CoA carboxylase (ACC), suppressing lipogenesis and enhancing fatty acid oxidation (FAO) [76]. Additionally, AMPK inhibits mTORC1 signaling, thereby modulating cell growth and proliferation. HFD consumption suppresses AMPK activity, leading to impaired energy homeostasis and increased lipid accumulation. Studies in HFD-fed mice demonstrate significant reductions in hepatic and skeletal muscle AMPK activity, resulting in increased triglyceride storage, insulin resistance, and mitochondrial dysfunction [77]. AMPK suppression also upregulates SREBP-1c expression, further promoting lipogenesis and exacerbating obesity-related metabolic dysfunction. In an HFD-induced obesity model, Garcia-Rodriguez et al. found that AMPK inhibition increased lipid droplet accumulation in hepatocytes, contributing to the progression of non-alcoholic fatty liver disease (NAFLD) [78]. Conversely, AMPK activators, such as metformin, restored lipid balance by enhancing FAO and reducing lipid-induced insulin resistance, highlighting the critical role of AMPK in mitigating HFD-induced metabolic disturbances [79].

2. SREBP-1c: The Lipogenesis Master Regulator and its Role in HFD-Induced Lipid Accumulation

SREBP-1c is a transcription factor that regulates de novo lipogenesis (DNL) by modulating the expression of lipogenic genes, including fatty acid synthase (FASN), acetyl-CoA carboxylase (ACC), and stearoyl-CoA desaturase-1 (SCD1). Activated by insulin and high lipid availability, SREBP-1c plays a crucial role in lipid storage and metabolism. HFDs significantly increase hepatic SREBP-1c activity, leading to enhanced triglyceride synthesis and lipid accumulation, contributing to NAFLD, insulin resistance, and obesity-related inflammation [80]. SREBP-1c overactivation also suppresses AMPK signaling, further exacerbating metabolic dysfunction [81]. A study found that HFD-fed mice exhibited increased hepatic SREBP-1c expression, resulting in excessive lipid accumulation and systemic metabolic dysregulation. Notably, SREBP-1c knockout mice were resistant to HFD-induced obesity and insulin resistance, suggesting that targeting SREBP-1c could mitigate metabolic dysfunction [82].

3. PPARγ: The Dual Role in Lipid Metabolism and Adipogenesis

PPARγ is a nuclear receptor that regulates adipogenesis, lipid storage, and insulin sensitivity. It is highly expressed in adipose tissue and liver, playing a critical role in lipid homeostasis. Its activation enhances fatty acid uptake and triglyceride storage, contributing to adipocyte expansion. HFD-induced obesity overactivates PPARγ, leading to increased lipid accumulation and systemic insulin resistance. While PPARγ activation promotes adipogenesis to buffer excess lipids, its sustained activation in HFD conditions exacerbates obesity-related inflammation and metabolic dysfunction [83]. Moussa et al. (2016) found that HFD-fed mice had increased PPARγ expression in adipose tissue, resulting in greater fat accumulation and impaired insulin signaling [84]. However, PPARγ antagonists, such as GW9662, reversed these effects, reducing lipid storage and improving insulin sensitivity [85].

4. Metabolic Pathway Effects of HFDs: AMPK, SREBP-1c, and PPARγ Interactions

HFDs profoundly alter metabolic pathways by modulating AMPK, SREBP-1c, and PPARγ, leading to systemic metabolic dysfunction. Suppression of AMPK activity reduces FAO while enhancing SREBP-1c-mediated DNL, promoting lipid accumulation. Increased SREBP-1c activity further drives hepatic steatosis, stimulating excessive fatty acid and triglyceride synthesis, which disrupts mitochondrial function and increases ROS production, contributing to NAFLD. Meanwhile, PPARγ overactivation promotes adipogenesis and lipid uptake, leading to obesity and sustained systemic insulin resistance and inflammation, further exacerbating metabolic dysfunction. Collectively, these disruptions create an imbalance between lipogenesis (SREBP-1c, PPARγ) and energy homeostasis (AMPK), fostering a pro-inflammatory, obesogenic environment that increases oxidative stress, accelerates HFD-induced cellular damage, and heightens the risk of cancer and metabolic disease progression.

### 3.4. Effect of HFDs on DDR: HFD-Induced DNA Damage and DDR Impairment

HFDs are strongly associated with the development of DNA damage and the attenuation of DDR pathways, which are essential for preserving genomic integrity. Research by Shimizu et al. shows that adipose tissue inflammation and systemic insulin resistance relate to obesity-associated DNA damage [86]. Obesity increasingly refers to inflammation, oxidative stress, and DNA damage. These studies explored the impact of the obesity setting on health outcomes by analyzing adipose tissue and peripheral blood mononuclear cells (PBMCs). They always found inflammation, metabolic dysregulation, and oxidative stress to be drivers of genomic instability. For instance, an Italian study in children reported increased nuclear-phosphorylated histone H2AX foci and micronuclei formation in PBMCs of overweight and obese individuals compared to normal-weight controls. These changes were associated with elevated circulating pro-inflammatory cytokines [53]. Similarly, patients diagnosed with metabolic syndrome experienced more frequent presence of DNA breaks and micronuclei in both blood and lymphocytes compared to their healthy counterparts, further supporting the idea of a systemic nature of metabolic diseases affecting genomic stability [87].

HFDs drive adipocyte hypertrophy through increased fatty acid influx, which stimulates excessive ROS production. Elevated ROS levels result in oxidative stress, causing lipid peroxidation, protein dysfunction, and nucleic acid oxidation, all contributing to genomic instability. Furukawa et al. reported that mice on HFDs showed significantly elevated levels of 8-hydroxy-2′-deoxyguanosine (8-OHdG), a biomarker of oxidative DNA damage, in both liver and adipose tissues. This elevation was strongly associated with increased ROS production and chronic inflammation, highlighting a direct relationship between HFD-induced oxidative stress and DNA damage [88]. Human studies further corroborate these findings, demonstrating that obese individuals have markedly higher levels of DNA damage markers, including γ-H2AX foci, which indicate double-strand breaks, compared to lean individuals [89].

These metabolic alterations induced by HFDs trigger genotoxic stress, thereby activating DDR pathways to repair lesions. The DNA repair system in eukaryotes, encompassing homologous recombination (HR), nonhomologous end joining (NHEJ), base excision repair (BER), and nucleotide excision repair (NER), is essential for maintaining genomic stability. DSBs are primarily repaired by HR and NHEJ, while single-strand breaks (SSBs) rely on BER and NER. However, the efficiency of DNA repair decreases with age, resulting in the accumulation of DNA damage in tissues [90]. This decline has been linked to age-related conditions such as metabolic and cardiovascular disorders, malignancies, and reduced lifespan, as demonstrated in studies involving both mice and humans with mutations in DNA repair genes [91,92].

Obesity-associated chronic inflammation plays a pivotal role in DDR impairment, primarily through sustained activation of the NF-κB signaling pathway. Pro-inflammatory macrophages, activated by obesity-related stress, secrete cytokines such as TNF-α and IL-6, which trigger the canonical NF-κB signaling cascade. In this pathway, ligand binding to TNF receptor (TNFR) or IL-6 receptor (IL-6R) activates the IKK complex (IKKα, IKKβ, and NEMO/IKKγ), which subsequently phosphorylates IκBα, marking it for degradation via the proteasome. This allows NF-κB (p65/p50 heterodimer) to translocate into the nucleus, where it binds to DNA and induces the transcription of pro-inflammatory genes (TNF-α, IL-6, IL-1β, COX-2, and NOS2), establishing a self-sustaining inflammatory loop [93,94]. Chronic NF-κB activation suppresses DDR by inhibiting the p53 pathway, a key DNA repair and apoptosis regulator. Specifically, NF-κB induces MDM2 expression, leading to p53 degradation via the ubiquitin–proteasome system, suppresses p53 transcription by recruiting co-repressors, and inhibits p53 target genes involved in cell cycle arrest and apoptosis [95]. This suppression allows DNA-damaged cells to evade apoptosis, increasing the risk of oncogenic transformation. Additionally, NF-κB suppresses ATM/ATR checkpoint signaling, impairing CHK1/CHK2 phosphorylation, which disrupts G2/M checkpoint arrest and prevents BRCA1/BRCA2 recruitment, thereby reducing HR repair efficiency [96,97]. These defects lead to the accumulation of DNA lesions, increasing genomic instability. Furthermore, NF-κB promotes mitochondrial dysfunction and NADPH oxidase (NOX) activation, resulting in excessive ROS generation, which induces oxidative DNA damage, including 8-oxo-guanine lesions, SSBs, and DSBs [98,99]. The suppression of BER and HR repair mechanisms further exacerbates genomic instability, promoting tumorigenesis. Moreover, systemic inflammation facilitates DNA damage beyond adipose tissue, as circulating cytokines activate resident macrophages in distant tissues, producing additional reactive species (COX-2, NOS, superoxide, and nitric oxide) that drive oxidative DNA damage [100,101,102]. Collectively, NF-κB-driven inflammation, DDR suppression, and oxidative stress create a pathological cycle that exacerbates DNA damage, promotes genomic instability, and increases cancer risk in obesity. Additionally, macrophages involved in clearing apoptotic cells migrate to other tissues, releasing factors that exacerbate DNA damage at these new sites [102]. This translocation concentrates damage in frequently infiltrated regions, including the gut, spleen, lymph nodes, and skin. Obesity-induced systemic inflammation and oxidative stress form an interconnected web of damage-inducing mechanisms that compromise genomic stability across multiple organs. Studies consistently show a strong association between obesity and elevated markers of DNA damage, such as γ-H2AX and 8-OHdG, in both metabolic and non-metabolic tissues. These findings underscore the extensive impact of HFDs on genomic integrity and their role in driving systemic health complications.

Obesity is closely linked to insulin resistance, which significantly disrupts DDR mechanisms through dysregulation of the PI3K/AKT/mTOR signaling pathway. Under normal physiological conditions, insulin binding to its receptor (IR) activates PI3K, leading to AKT phosphorylation, which regulates cell survival, proliferation, and metabolism [103]. However, in obesity-induced hyperinsulinemia, chronic activation of AKT suppresses FOXO transcription factors (FOXO1, FOXO3a, FOXO4, and FOXO6), which are essential for oxidative stress response and DNA repair [104]. FOXO inhibition reduces the expression of genes involved in NER and HR, leading to increased oxidative stress and genomic instability [105]. Additionally, persistent activation of mTOR complex 1 (mTORC1), a downstream effector of AKT, inhibits autophagy, preventing the clearance of damaged mitochondria and proteins. This exacerbates ROS accumulation, further compromising genomic integrity [106]. This metabolic dysfunction also promotes p53 degradation, impairing p53-dependent apoptosis and DNA repair, allowing genetically unstable cells to evade cell cycle arrest [107]. Overactivation of PI3K/AKT/mTOR signaling disrupts ATM/ATR checkpoint activation, leading to defects in CHK1/CHK2-mediated checkpoint signaling and impairing DDR pathways such as HR and NHEJ, increasing chromosomal aberrations and mutation rates [108].

This DDR impairment, coupled with metabolic stress and chronic inflammation, fosters a pro-tumorigenic environment, elevating the risk of obesity-associated cancers, including colorectal, breast, pancreatic, and liver cancer [109,110].

The dysregulation of DDR is further linked to a spectrum of metabolic disorders, underscoring the critical role of DNA repair in maintaining genomic stability and metabolic homeostasis. Recent studies identified key DNA repair factors that not only guard against genomic instability, but also regulate cellular metabolism following DNA damage [111,112,113]. In obesity, chronic inflammation and oxidative stress place a substantial burden on DDR machinery, leading to the accumulation of DNA lesions, such as DSBs and base modifications, that saturate repair pathways. The compromised efficiency of DDR systems in obesity is closely associated with disrupted glucose metabolism, further amplifying metabolic dysfunction and systemic imbalance.

In response to oxidative DNA damage, the tumor suppressor protein p53 plays a central role in regulating cell cycle arrest, senescence, and apoptosis to maintain genomic integrity. Tornovsky-Babeay et al. demonstrated that excessive glucose metabolism intensifies DNA damage by generating DSBs, triggering p53 activation and apoptosis via oxidative stress pathways [114]. Chronic HFD consumption further exacerbates this scenario by overwhelming DDR mechanisms, impairing DNA repair efficiency, and increasing genomic instability. Moreover, p53 exerts a dual role in adipose tissue, contributing to both inflammation and metabolic dysregulation. Minamino et al. demonstrated that p53 overexpression in adipose tissue led to localized inflammation, impaired insulin signaling, and systemic glucose dysregulation [115]. In HFD-fed mice, sustained p53 activation intensified the release of pro-inflammatory cytokines, including IL-6 and TNF-α, further aggravating insulin resistance and metabolic dysfunction [116,117]. These findings underscore the intricate interplay between DDR impairment, oxidative stress, and metabolic dysregulation in obesity and highlight the necessity for targeted therapeutic strategies to restore DDR efficiency and mitigate obesity-driven cancer risks.

Obesity exacerbates disruptions in DDR pathways. For instance, an inverse relationship has been identified between body mass index (BMI) and NER capacity, particularly in young females [118]. Obesity has also been associated with deficiencies in repairing double-strand breaks (DSBs) caused by genotoxic agents [119]. Elevated ROS levels in obesity alter the expression of genes vital for DNA repair [120,121] and suppress the activity of DNA repair enzymes, thereby exacerbating genomic instability [111]. Moreover, obesity-related metabolic changes influence genes involved in cellular stress responses and detoxifying harmful agents [122]. Emerging research has shown that epigenetic maintenance is essential for regulating DNA repair genes, which can be disrupted due to HFDs and unbalanced nutrition. These diets and nutrition patterns can change the methyl groups available for ribosomal production and usage. These alterations cause aberrant DNA methylation patterns and changes in gene expression profiles [123]. Antioxidant interventions, such as epigallocatechin-3-gallate (EGCG), demonstrated the potential to counteract these effects. For instance, EGCG has been shown to enhance the methylation and expression of key DNA repair genes, including MGMT and MLH1, in obese mice [124]. In contrast, data show that HFD-induced expression loss of MLH1 and higher methylation rates are associated with increased DNA damage and suggest an essential function of epigenetic regulation in DDR [125]. They show how diet, oxidative stress, and epigenetic alterations affect DDR’s efficiency.

### 3.5. Overview of HFD-Induced Metabolic Syndrome and Its Impact on Radiation Response

HFDs are a key contributor to the global rise in metabolic syndrome, a condition characterized by obesity, insulin resistance, dyslipidemia, and chronic low-grade inflammation. These dietary patterns disrupt metabolic and cellular homeostasis, with consequences that extend beyond metabolic health to influence cellular stress responses, including the DNA damage response and sensitivity to radiation. HFDs play an instrumental role in the development of metabolic syndromes such as diabetes and cardiovascular disease, where obesity, insulin resistance, dyslipidemia, and chronic low-grade inflammation constitute the four-finger markers of the disease. Excessive intake of dietary saturated fats disturbs cellular homeostasis and systemic metabolic functions, resulting in metabolic disruptions. A common feature of HFD-induced metabolic syndrome is increased circulating FFAs, which drive ectopic fat deposition in tissues, including the liver, skeletal muscle, and pancreas. This lipid accumulation stimulates lipotoxicity, mitochondrial dysfunction, and oxidative stress, impairing glucose metabolism and promoting insulin resistance [33]. Metabolic syndrome is characterized by chronic low-grade inflammation caused by macrophages infiltrating into adipose tissue. Macrophages in obese adipose tissue undergo a shift to a pro-inflammatory (M1) phenotype, secreting cytokines such as TNF-α and IL-6. These cytokines disrupt insulin signaling by modifying insulin receptor substrates and impair glucose uptake [36]. Oxidative stress exacerbates the inflammatory state by generating ROS, damaging lipids, proteins, and nucleic acids. The interplay between oxidative stress and inflammation drives the progression of metabolic syndrome and associated diseases such as type 2 diabetes and NAFLD [117].

The metabolic disturbances induced by HFDs have profound implications for radiation response due to shared pathways involving oxidative stress, inflammation, and DNA damage. IR produces ROS directly and indirectly, causing oxidative damage to cellular components, including DNA. Kept in a state of cellular damage from HFD-induced metabolic syndrome, with increased basal ROS levels and chronic inflammation, individuals will have cells that are “preconditioned” to be more sensitive to damage from radiation. This increased oxidative stress leads to increased production of radiation-induced ROS, further contributing to DNA damage and cellular dysfunction [25]. HFDs also compromise DNA damage response, a critical mechanism for the maintenance of genomic stability involving radiation exposure. Chronic oxidative stress and inflammation from HFDs overwhelm DDR pathways, reducing the efficiency of key DNA repair processes. Studies have shown that obesity and high BMI correlate with reduced DDR capacity, increasing susceptibility to radiation-induced genomic instability [126]. Epigenetic modifications, such as changes in DNA methylation, further disrupt DDR by altering the expression of DNA repair genes [127].

The combination of HFD-induced metabolic syndrome and radiation exposure significantly increases the risk of adverse clinical outcomes, including secondary malignancies, delayed wound healing, and diminished efficacy of radiation therapy in cancer patients. HFD-driven chronic inflammation and oxidative stress foster a pro-inflammatory tumor microenvironment (TME), contributing to tumor resistance to radiation. A key hallmark of HFD-induced metabolic syndrome is mitochondrial dysfunction, which compromises cellular energy reserves and impairs antioxidant defense mechanisms, further reducing the body’s ability to counteract radiation-induced damage. Epidemiological studies extensively documented the association between HFD consumption, obesity, and increased cancer incidence. Large-scale cohort studies and meta-analyses consistently reported a strong correlation between diets high in saturated fats and refined carbohydrates with elevated cancer risk across diverse populations. For example, the European Prospective Investigation into Cancer and Nutrition (EPIC) study, which tracked over 500,000 individuals, found that high dietary fat intake was significantly associated with an increased risk of colorectal and breast cancer [128]. Similarly, findings from the Nurses’ Health Study (NHS) and the Health Professionals Follow-Up Study (HPFS) demonstrated that long-term HFD consumption was linked to a higher incidence of pancreatic and prostate cancer, with obesity serving as a key mediator [129]. Furthermore, a meta-analysis of 203 studies involving more than 6.3 million participants indicated that obesity and HFDs were associated with increased mortality in cancer patients, particularly those with postmenopausal breast cancer, uterine cancer, and colorectal cancer [130]. These studies highlight the necessity of addressing HFD-related metabolic alterations as modifiable risk factors for cancer prevention and improved patient outcomes.

Beyond radiation resistance, HFDs promote cancer cell survival through obesity-driven hyperinsulinemia and chronic inflammation, which activate oncogenic pro-survival pathways, including PI3K/AKT and STAT3. These pathways enhance cellular proliferation, suppress apoptosis, and facilitate tumor progression. Hyperinsulinemia, a common metabolic consequence of obesity, leads to sustained activation of the IGF-1R, which in turn stimulates PI3K/AKT signaling. This cascade results in enhanced tumor cell survival and resistance to therapy by promoting proliferation and inhibiting pro-apoptotic proteins such as BAD and BIM [131]. Similarly, STAT3 activation by inflammatory cytokines such as IL-6 and TNF-α establishes an oncogenic feedback loop by upregulating anti-apoptotic proteins (e.g., Bcl-2, Mcl-1), promoting immune evasion, and facilitating epithelial–mesenchymal transition (EMT), which further drives tumor progression and metastasis [132].

HFDs also enhance cancer cell survival by promoting lipid accumulation, metabolic adaptation, and chemoresistance. Excessive lipid storage in tumors provides an alternative energy source that enhances metabolic flexibility, allowing cancer cells to thrive in nutrient-deprived conditions. Studies have shown that lipid-enriched cancer cells exhibit increased expression of fatty acid oxidation (FAO) enzymes, such as carnitine palmitoyltransferase 1A (CPT1A) and acyl-CoA synthetase long-chain family member 4 (ACSL4), which sustain ATP production and confer resistance to apoptosis [133]. Additionally, lipid peroxidation byproducts, such as 4-hydroxynonenal (4-HNE), activate NRF2, a key regulator of redox homeostasis, enabling tumor cells to mitigate oxidative stress induced by radiation and chemotherapy [134]. Lipid rafts in tumor cell membranes serve as structural platforms for the activation of pro-survival kinases, including AKT and Src, further reinforcing resistance to therapy [135]. Clinical studies consistently link increased lipid metabolism to poor prognosis across multiple cancers, highlighting HFD-mediated metabolic reprogramming as a key factor in tumor survival and therapy resistance [136].

Beyond direct effects on cancer cells, HFDs profoundly alter the TME by modulating macrophage function. Obesity-associated chronic inflammation, driven by HFD-induced metabolic dysregulation, leads to increased infiltration of tumor-associated macrophages (TAMs) into the TME. TAMs, particularly those polarized toward the M2 phenotype, secrete immunosuppressive cytokines such as IL-6, IL-10, and TGF-β, which suppress anti-tumor immunity and promote cancer cell proliferation, survival, and metastasis [137]. Furthermore, HFDs exacerbate lipid accumulation within TAMs, altering their metabolic state and reinforcing their immunosuppressive properties, further fueling tumor progression. Nieman et al. demonstrated that obesity-driven lipid availability fuels macrophage-mediated inflammation, leading to cancer cell survival through NF-κB activation and the upregulation of anti-apoptotic genes such as Bcl-2 and Mcl-1 [138]. This inflammation-induced resistance facilitates tumor growth and significantly reduces the efficacy of therapeutic interventions, including chemotherapy and radiation therapy.

Given the strong association between HFDs, obesity, and cancer, dietary and lifestyle interventions hold promise for lowering cancer risk and improving patient outcomes. Nutritional interventions, such as the adoption of a Mediterranean diet rich in unsaturated fats, fiber, and polyphenols, have been shown to reduce obesity-related inflammation and improve metabolic health [139]. Additionally, intermittent fasting and caloric restriction strategies demonstrated efficacy in lowering insulin resistance, reducing IGF-1 signaling, and enhancing the effectiveness of cancer therapies [140]. Beyond lifestyle modifications, targeted metabolic therapies offer promising approaches to counteract HFD-induced cancer progression. mTOR inhibitors (e.g., rapamycin) and AKT inhibitors have been proposed to restore DDR efficiency and suppress tumor growth in obesity-driven cancers [141]. Furthermore, pharmacological activation of FOXO transcription factors has shown potential in reversing HFD-induced metabolic dysfunction and increasing cancer cell sensitivity to chemotherapy [142]. Given that obesity exacerbates DNA damage and impairs repair mechanisms, antioxidant-based interventions, such as polyphenol-rich compounds (e.g., epigallocatechin-3-gallate), may counteract oxidative stress and improve genomic stability in high-risk populations [143].

The cumulative evidence highlights the profound impact of HFDs on cancer biology, where metabolic reprogramming, chronic inflammation, and oncogenic signaling converge to drive tumor resistance to radiation and therapeutic interventions. Understanding these dysregulated pathways is critical for developing targeted strategies to mitigate the oncogenic effects of HFDs in cancer patients. Given the strong link between HFD consumption and tumor progression, interventions targeting lipid metabolism, macrophage polarization, and pro-survival signaling pathways such as PI3K/AKT and STAT3 may offer promising therapeutic avenues to enhance cancer treatment outcomes in obese individuals. Targeted interventions, including dietary modifications, antioxidant therapies, and inflammation-targeted treatments, hold great potential in mitigating the detrimental effects of HFDs on cancer progression and radiation response.

## 4. IR and HFDs

Excessive exposure to IR and prolonged consumption of HFDs are both associated with significant health risks, including metabolic disturbances such as obesity. Interestingly, under specific conditions, IR may mitigate the metabolic effects induced by HFD consumption. Conversely, HFDs can exacerbate the detrimental effects of IR exposure. This section systematically examines these interactions, focusing on three key outcomes: IR-induced obesity, the amelioration of HFD-induced metabolic disturbances by IR, and the amplification of IR-induced harm by HFDs. By elucidating the underlying mechanisms, this section aims to offer insights for refining radiation risk assessments by integrating lifestyle factors such as dietary habits.

### 4.1. IR-Induced Obesity and Metabolic Dysregulation

#### 4.1.1. Overview

Exposure to IR elicits a wide range of health effects on the human body, encompassing both immediate and long-term consequences. These effects have been extensively documented through clinical observations, epidemiological studies, and experimental research. These studies underscore the significant health risks associated with IR exposure, including the development of metabolic disturbances such as obesity [144,145]. Among these outcomes, increasing evidence highlights IR as a contributing factor to metabolic dysfunction, particularly in the context of obesity and related disorders. While numerous studies established a link between IR exposure and metabolic alterations, such as the onset of obesity and related disorders [146,147], other studies suggest that IR may induce metabolic dysfunction without necessarily causing increased fat accumulation. For example, sublethal whole-body irradiation (WBI) in nonhuman primates led to diabetes mellitus and insulin resistance in adipose tissue, but did not induce obesity, indicating that IR can drive metabolic diseases independently of fat accumulation [148].

Notably, the metabolic changes induced by IR bear similarities to those associated with HFDs, including adiposity, lipid accumulation, and obesity. Long-term follow-up studies of atomic bomb survivors provided critical insights into the relationship between IR exposure and metabolic risk factors such as lipid accumulation and fatty liver, which are precursors to obesity [146]. Recent studies further emphasized the role of IR-induced oxidative stress, inflammation, and endocrine dysregulation in contributing to metabolic syndrome and obesity [147]. Experimental research supports these findings, with studies in B6C3F1 mice showing dose-dependent weight gain following continuous low-dose-rate gamma irradiation. Male mice exposed to 1.1 mGy/day and female mice exposed to 21 mGy/day exhibited significant weight gain between 32 and 60 weeks of age, implicating IR in metabolic disturbances independent of dietary factors [149]. Similarly, exposure to 20 mGy/day of IR increased adiposity, liver lipid content, and serum leptin levels without a corresponding increase in food intake, suggesting a direct effect of IR on lipid metabolism [150].

Thoracic region irradiation (TRI) at 12.5 Gy in female C57BL/6 mice resulted in progressive obesity, with 42.1% of the animals becoming overweight within four months and 100% within ten months, accompanied by increased abdominal fat accumulation and disruptions in lipid metabolism, paralleling the outcomes of HFD exposure [151]. Similarly, minipig models exposed to 1.9–2.0 Gy X-rays exhibited systemic metabolic dysregulation, including altered lipid profiles, decreased circulating insulin-like growth factor I (IGF-1) levels, and disrupted cardiac IGF-1 signaling. These findings link IR exposure to metabolic syndrome and endocrine dysfunction, mirroring the pathophysiological effects observed with HFDs [152]. Collectively, these studies underscore the significant impact of IR on metabolic processes, highlighting shared pathophysiological mechanisms between IR-induced metabolic disorders and HFD-induced obesity.

#### 4.1.2. Mechanisms

IR-induced obesity (IRIO) and metabolic dysregulation result from complex disruptions in metabolic, mitochondrial, and hormonal pathways. Chronic exposure to IR triggers dose-dependent weight gain by disrupting lipid regulation, energy balance, and insulin signaling. In subcutaneous (SQ) adipose tissue, IR exposure leads to reduced phosphorylation of insulin receptor substrate-1 (IRS-1), reflecting impaired insulin signaling, along with increased basal lipolysis, contributing to a dysfunctional adipose environment. Enlarged adipocytes, or hypertrophy, are also observed, which further impairs glycemic control. Additionally, increased macrophage infiltration in irradiated SQ adipose tissue correlates with reduced levels of adiponectin, a key anti-inflammatory and insulin-sensitizing hormone, suggesting that inflammation plays a central role in the development of metabolic dysfunction. Notably, there is no evidence of fibrosis in SQ adipose tissue, suggesting that these dysfunctions are not driven by fibrosis [148].

Mitochondrial dysfunction is a pivotal aspect of IRIO, as evidenced by decreased mitochondrial DNA (mtDNA) copy numbers and increased mtDNA mutations in key organs, such as the heart and liver. These mitochondrial impairments compromise energy homeostasis and promote fat storage [151]. These disruptions are compounded by increased adiposity, elevated liver and serum lipid content, and higher leptin levels, all indicative of impaired adipocyte signaling and systemic lipid accumulation. Importantly, these metabolic alterations occur independently of increased food intake, highlighting the direct influence of IR on lipid metabolism [150].

Hormonal disturbances also play a crucial role in IRIO. Reduced expression of carnitine palmitoyltransferase 1 (CPT1) and insulin receptor substrate-2 (IRS-2), both essential for lipid metabolism and insulin sensitivity, have been observed. Furthermore, dysregulation of IGF-1 signaling emerged as a central mechanism, with decreased circulating IGF-1 levels and impaired cardiac IGF-1 signaling contributing to obesity, insulin resistance, and systemic metabolic syndrome. Chronic IR exposure exacerbates tissue damage and fibrosis, mediated by elevated transforming growth factor-beta-1 (TGFβ-1) levels, which impair organ function and exacerbate metabolic and cardiovascular abnormalities [152]. These findings illustrate how mitochondrial dysfunction, hormonal disturbances, and metabolic alterations induced by IR converge to promote obesity and its associated complications [149,150,151,152]. Table 1 summarizes the mechanisms of IR-induced obesity and metabolic dysregulation across organ systems.

#### 4.1.3. Summary

Sublethal WBI in male rhesus macaques induces significant metabolic disturbances, including diabetes mellitus and insulin resistance in subcutaneous adipose tissue, without leading to obesity. Key findings highlight impaired insulin signaling, increased lipolysis, macrophage-driven inflammation, and adipocyte hypertrophy, which collectively impair glycemic control and overall metabolic function. Importantly, dysfunction in subcutaneous adipose tissue, rather than obesity or fibrosis, emerges as the primary driver of IR-induced metabolic disease in nonhuman primates.

Similarly, chronic low-dose-rate IR exposure results in IRIO, with notable metabolic and systemic disruptions. In B6C3F1 mice, prolonged gamma radiation exposure at varying dose rates led to dose-dependent weight gain and the development of neoplasms, such as myeloid leukemia and granulosa cell tumors, suggesting both oncogenic and metabolic consequences. IR-induced adiposity and lipid accumulation in the liver and serum, independent of food intake, were associated with disruptions in lipid metabolism and adipocyte signaling, including elevated leptin levels. TRI in female C57BL/6 mice produced obesity similar to that induced by HFDs, driven by mitochondrial dysfunction, reduced mtDNA copy numbers, increased mtDNA mutations, and downregulation of lipid regulators such as CPT1 and IRS2. In Göttingen minipigs, IR exposure led to systemic metabolic abnormalities, including altered lipid profiles, reduced circulating IGF-1, and tissue fibrosis, linking IR exposure to metabolic syndrome and cardiovascular dysfunction. Collectively, these studies highlight the complex mechanisms underlying IRIO, including mitochondrial dysfunction, hormonal dysregulation, and systemic metabolic disruptions, and underscore the significant health implications of IR exposure.

These findings emphasize the intricate mechanisms by which IR induces metabolic disturbances, encompassing mitochondrial dysfunction, hormonal imbalances, and systemic metabolic changes. They highlight the urgent need for further research to fully elucidate these mechanisms and develop strategies to mitigate the long-term metabolic and systemic effects of IR exposure.

### 4.2. IR-Induced Alleviation of HFD-Induced Metabolic Effects

#### 4.2.1. Overview

Experimental evidence increasingly supports the potential of IR in mitigating metabolic dysfunction associated with HFDs and diabetes. Early studies demonstrated that localized high-dose irradiation using Iridium-192 wire (11–14 Gy) significantly reduced atherosclerotic lesions in cholesterol-fed rabbits, particularly in irradiated aortic regions compared to controls [153,154]. Moreover, repeated low-dose radiation (LDR) exposure has been demonstrated to alleviate diabetes-induced renal dysfunction by improving key renal biomarkers, including decreased urinary microalbumin and increased creatinine levels, while simultaneously preventing fibrosis and oxidative damage. Among various exposure regimens, WBI with 12.5 mGy X-rays administered every other day for 8 weeks was identified as the most effective in providing renal protection [155,156]. Furthermore, LDR protocols (50 and 75 mGy for 4 weeks) in diabetic mouse models significantly improved dyslipidemia, insulin resistance, and kidney pathology [157,158].

Fractionated whole-body gamma irradiation (FWBGI), delivered at low doses (0.5–2 Gy, administered three times weekly for two months), was observed to mitigate HFD-induced hepatic inflammation and promote anti-diabetic gene expression, indicating systemic metabolic benefits [159,160,161]. Similarly, LDR exposure has been shown to inhibit atherosclerosis by reducing neutrophil extracellular traps and plaque formation in atherosclerosis models induced by HFD [162]. Additionally, IR has been found to modulate lipid metabolism, with HFDs conferring partial resistance to IR-induced skin injuries, likely due to alterations in lipid remodeling processes [163].

Collectively, these studies highlight the diverse and promising applications of IR in the management of metabolic diseases and their associated complications, primarily through mechanisms involving inflammation suppression, lipid metabolism modulation, and enhanced insulin sensitivity.

#### 4.2.2. Mechanisms

IR mitigates HFD-induced metabolic dysfunction through diverse mechanisms including suppression of inflammation and fibrosis, improved insulin sensitivity and glucose metabolism, lipid metabolism modulation, and mitochondrial regulation. Early studies revealed that high-dose localized IR reduced atherosclerotic lesions in hypercholesterolemic rabbit aortas by suppressing the intimal hyperplastic response, a pathological process triggered by chronic hypercholesterolemia [153,154]. Recent research highlighted the systemic and renal benefits of LDR, particularly in alleviating diabetes-induced kidney damage. These protective effects are mediated by reduced oxidative damage, suppression of inflammatory markers (e.g., TNFα, IL-18, and MCP-1), and inhibition of fibrosis through downregulation of connective tissue growth factor and fibrotic markers, such as collagen IV and fibronectin [155,156,157]. LDR also improves systemic metabolic parameters, including dyslipidemia and insulin resistance, through mechanisms such as enhanced protein kinase B activation and Nrf2-mediated antioxidant responses, although its efficacy is limited by a defined therapeutic window [158].

FWBGI has shown analogous benefits, including the suppression of pro-inflammatory cytokines (e.g., TNFα, IL-1β, and IL-6) and the promotion of an anti-inflammatory balance by upregulating IL-10 expression [159]. FWBGI also enhances glucose metabolism by increasing glucagon-like peptide-1 (GLP-1) expression across intestinal segments and promoting insulin receptor substrate-4 and uncoupling protein 3 (UCP3) expression in the colon, thereby improving insulin sensitivity and energy expenditure [160]. Furthermore, FWBGI modulates systemic pathways through the upregulation of GLP-1 receptors (GLP-1Rs) in the liver and brain and activates mitochondrial uncoupling of proteins 2 and 3 (UCP2/3), which reduce oxidative stress and improve energy metabolism. These findings suggest a coordinated interaction between the brain, liver, and intestine in mitigating HFD-induced metabolic dysfunction [161].

In addition to its systemic benefits, IR exposure modulates lipid metabolism and promotes tissue repair. Specifically, it downregulates key lipid metabolic pathways while facilitating keratinocyte and fibroblast migration, which is crucial for tissue regeneration. Palmitic acid and fatty acid-binding protein 4 (FABP4) play pivotal roles in DNA damage repair and radioprotection in irradiated tissues [162]. Moreover, LDR inhibits the formation of neutrophil extracellular traps (NETs), a major contributor to HFD-induced atherosclerosis, thereby reducing endothelial plaque formation. This effect is associated with the modulation of immune pathways, emphasizing the central role of neutrophils in the progression of atherosclerosis [163].

Collectively, IR alleviates HFD-induced metabolic dysfunction through diverse mechanisms, including suppression of inflammation, oxidative stress, and fibrosis; enhancement of insulin sensitivity; modulation of lipid metabolism; and attenuation of atherosclerosis-related processes. These findings underscore its potential as a multifaceted therapeutic intervention for metabolic diseases and their associated complications. Table 2 summarizes the mechanisms of IR-induced alleviation of HFD-induced metabolic effects across organ systems.

#### 4.2.3. Summary

IR demonstrates significant potential in mitigating metabolic dysfunction and complications induced by HFD. Localized IR effectively suppresses atherogenesis by reducing intimal hyperplasia associated with chronic hypercholesterolemia, thereby attenuating the progression of cholesterol-induced atherosclerosis in rabbit models. LDR has shown renoprotective effects in diabetic models by mitigating oxidative stress, inflammation, and fibrosis, which collectively enhance renal function and structural integrity. Optimized LDR regimens and their combination with fibroblast growth factor-21 (FGF21) further amplify these systemic benefits by addressing key factors such as dyslipidemia, insulin resistance, and diabetes-induced renal pathology. FWBGI demonstrated anti-inflammatory properties in HFD-induced liver inflammation, improving the balance between pro- and anti-inflammatory cytokines. It also prevents pre-diabetic conditions by enhancing GLP-1 expression, improving glucose metabolism, and activating colon-specific metabolic pathways, such as IRS-4 and UCP3. Furthermore, FWBGI modulates mitochondrial regulation by increasing GLP-1 receptor (GLP-1R) expression and UCP2/3 activity in the liver and brain, suggesting a novel regulatory axis involving the brain, liver, and intestines for managing type 2 diabetes. IR also plays a role in lipid metabolism modulation, where adipocytes contribute to tissue repair and DNA damage mitigation through FABP4 and palmitic acid. Additionally, LDR inhibits the release of NETs, a key driver of atherosclerosis, thereby reducing plaque formation in HFD-induced cardiovascular models.

These findings underscore the diverse applications of IR in alleviating HFD-induced metabolic disorders and highlight its potential for innovative radiotherapy-based interventions targeting systemic and organ-specific pathologies.

### 4.3. Enhancement of IR-Induced Detrimental Effects by HFDs

#### 4.3.1. Overview

HFDs significantly exacerbate the detrimental effects of IR through synergistic mechanisms that impact survival, vascular health, metabolic function, and systemic inflammation. Early studies from the 1960s demonstrated that HFDs elevate serum cholesterol levels, disrupt lipid metabolism by increasing LDL and decreasing high-density lipoprotein (HDL), and induce atherosclerotic lesions in major arteries, such as the thoracic and abdominal aorta, in the absence of IR exposure [164,165,166]. When combined with IR, HFDs accelerate the progression and severity of atherosclerosis, particularly in vulnerable regions such as the coronary and pulmonary arteries, by promoting oxidative stress, inflammation, and lipid deposition at sites of IR-induced arterial injury [167,168,169]. IR exposure enhances the proliferation of smooth muscle cells (SMCs), increasing their responsiveness to cholesterol and contributing to more extensive atherosclerotic lesions. Chronic IR to low-dose gamma or neutron irradiation further intensifies arterial inflammation and tissue damage, amplifying the harmful effects of HFDs [168,170]. In addition to cardiovascular effects, HFDs modulate metabolic, epigenetic, and immune responses, amplifying IR-induced damage across various biological systems. HFD-induced obesity alters IR-triggered epigenetic mechanisms, including promoter methylation of key genes, and disrupts microRNA (miR) regulation, such as the upregulation of miR-466e, which enhances radiosensitivity [171]. Obese mice exhibit a diminished adaptive response to IR, with significantly reduced 30-day survival following WBI and a lethal dose 50 (LD50) of 6.0 Gy, compared to 7.1 Gy in mice on a standard diet [172]. Furthermore, HFDs fail to mitigate IR-induced genotoxicity, as evidenced by the persistent presence of micronucleated erythrocytes in bone marrow, and exacerbate metabolic dysfunctions, including insulin resistance and disrupted glucose metabolism in skeletal muscle and adipose progenitor cells [173]. In the liver, the combination of HFD and IR potentiates IR-induced fibrosis and cancer risk, suggesting that dietary factors contribute to increased susceptibility to liver damage from radiation [174]. Obesity further amplifies IR-induced damage to hematopoietic stem and progenitor cells (HSPCs), reducing their populations and promoting the survival of leukemia blasts, likely through alterations in the bone marrow microenvironment [175]. Co-exposure to HFD and LDR exacerbates metabolic impairments, insulin resistance, and intestinal barrier dysfunction, with more severe effects observed in female mice [176,177]. Notably, sex-specific differences are evident, with female mice on HFD showing more severe intestinal injury and increased radiosensitivity compared to male mice. Melatonin supplementation has been shown to mitigate hematopoietic damage and promote intestinal recovery following whole abdominal irradiation (WAI) [13]. Additionally, alterations in gut microbiota induced by HFD further exacerbate radiation-induced damage, reducing microbial diversity, increasing systemic inflammation, and amplifying both local and systemic radiation effects [12]. Moreover, HFD synergizes with LDR to worsen vascular stiffness, lipid dysregulation, and inflammatory responses, thereby accelerating the development of atherosclerosis in ApoE−/− mice [178].

Collectively, these findings underscore the role of HFDs in amplifying IR-induced damage and highlight the importance of dietary and therapeutic interventions to mitigate the compounded health risks associated with concurrent exposure.

#### 4.3.2. Mechanisms

The combination of HFDs and IR leads to synergistic effects that exacerbate IR-induced damage through multiple mechanisms, including metabolic dysregulation, oxidative stress, inflammation, and epigenetic changes. HFDs independently induce metabolic imbalances, such as elevated LDL levels, reduced HDL levels, and increased serum cholesterol, which predispose the vasculature to lipid deposition and oxidative stress, even in the absence of IR exposure [164,166]. When combined with IR, HFDs amplify vascular injury by exacerbating lipid deposition in damaged arterial walls, enhancing inflammation and further activating oxidative stress pathways already initiated by the diet [165,168,170]. This interaction accelerates atherosclerosis, particularly in vulnerable regions such as the coronary and pulmonary arteries, through mechanisms such as SMC proliferation and increased responsiveness to cholesterol, resulting in more extensive and severe atherosclerotic lesions [167,179]. The exacerbation of atherosclerosis is not merely the additive effect of the two insults, but a result of complex metabolic and cellular responses, including transient DNA synthesis in SMCs, which further contribute to lesion severity [179]. Recent studies further elucidated the role of epigenetic modifications, oxidative stress, and gut microbiota dysregulation in this synergistic relationship. HFD-induced DNA methylation changes in critical gene promoters (e.g., p16, Dapk, Mgmt, Ep300, and Socs1) and the disruption of microRNA regulation, such as the upregulation of miR-466e, have been implicated in radiosensitization, particularly in the presence of free fatty acids [171]. Chronic oxidative stress associated with obesity exacerbates these alterations, impairing the adaptive response to IR and reducing protection against WBI, as indicated by elevated levels of micronucleated erythrocytes in bone marrow [172]. In skeletal muscle and adipose progenitor cells, HFD-induced epigenetic reprogramming disrupts intracellular signaling pathways, leading to persistent dysregulation of insulin signaling even after cellular differentiation [173]. The liver, due to its high metabolic activity, exhibits heightened susceptibility to IR-induced damage, with HFD further amplifying oxidative stress, inflammation, fibrosis, and cancer risk [174]. Obesity also intensifies IR-induced suppression of HSPCs, elevates ROS levels, and alters the bone marrow microenvironment, creating conditions that support leukemia blast survival and proliferation [175]. Co-exposure to HFD and LDR induces significant alterations in gut microbiota composition, including an increase in parasutterella abundance and elevated pyrrolidinecarboxylic acid (PA) levels. PA enhances pyrroline-5-carboxylate reductase 1 (PYCR1) expression, which inhibits the protein kinase b/mammalian target of rapamycin (Akt/mTOR) insulin signaling pathway, further aggravating metabolic dysfunction [177]. Additionally, HFD combined with WAI worsens intestinal damage, reduces microbial diversity, and increases systemic inflammation, with pronounced sex-specific differences, particularly in female mice [13,177]. Melatonin supplementation has been shown to restore intestinal stem cells, modulate gut microbiota, and ameliorate HFD- and IR-induced damage in a sex-specific manner [13]. Furthermore, LDR uniquely activates the cyclic GMP-AMP synthase stimulator of the interferon genes (cGAS-STING) signaling pathway by increasing cytosolic mitochondrial DNA and cGAS protein expression. This activation triggers the release of type I interferons (IFN-α/β), amplifying inflammatory responses and promoting atherosclerosis progression. When combined with HFD-induced lipid dysregulation, these inflammatory effects accelerate vascular damage and plaque formation [178]. Together, these findings underscore the significant impact of HFD on IR-induced biological responses and highlight the need for targeted dietary and therapeutic strategies to mitigate the compounded effects of these factors and improve health outcomes. Table 3 summarizes the mechanisms underlying enhancement of IR-induced detrimental effects by HFDs across organ systems.

#### 4.3.3. Summary

Early investigations into the synergistic effects of HFDs and IR on cardiovascular health, particularly atherosclerosis, demonstrated that HFDs alone induce lipid metabolism disturbances, elevated serum cholesterol, and the development of atherosclerotic lesions in major arteries. However, concurrent exposure to IR, whether with X-rays, gamma rays, or neutron radiation, exacerbates these effects, resulting in more severe vascular damage, increased lipid deposition, and inflammation. For instance, studies have shown that IR increases the susceptibility of arterial walls to dietary cholesterol, accelerating plaque development and arteriosclerosis, especially in the coronary and pulmonary arteries. These interactions are driven by oxidative stress, disrupted lipid metabolism, and enhanced inflammatory markers, such as vascular adhesion molecules. Notably, these effects are not solely due to direct IR-induced DNA damage in smooth muscle cells, but involve complex biochemical pathways that promote lipid accumulation and vascular injury. Additionally, the type and level of dietary fat, such as cottonseed oil or margarine, significantly influence survival rates in irradiated mice, highlighting the role of fat composition in modulating IR outcomes. Recent studies further elucidated how HFDs modulate the biological effects of IR through epigenetic mechanisms, such as changes in gene promoter methylation, and disruption of microRNA regulation, particularly miR-466e, which plays a significant role in radiosensitization. In murine models, HFDs impair the adaptive response to IR, leading to reduced survival rates following WBI, increased susceptibility to IR-induced genotoxic effects, as well as decreased capacity to mitigate IR-induced genomic instability. HFDs also exacerbate metabolic dysfunctions, such as insulin resistance and hyperinsulinemia, through epigenetic modifications and disrupted signaling in metabolic progenitor cells, thus increasing the risk of chronic metabolic diseases. The liver, due to its high metabolic activity, is particularly vulnerable to radiation-induced conditions, including fibrosis and cancer, with these risks further amplified by HFDs that disrupt normal regulatory mechanisms. Additionally, obesity exacerbates radiation-induced damage to the hematopoietic system by depleting HSPC populations and enhancing the survival of leukemia blasts, likely mediated by oxidative stress and alterations in the bone marrow microenvironment. Conversely, exercise has been shown to mitigate these effects by restoring HSPC populations, reducing ROS, and improving bone marrow functionality, thus offering a potential therapeutic strategy for cancer survivors. The combination of HFD and LDR also exacerbates metabolic dysfunction by disrupting insulin signaling through the PA-PYCR1 axis and damaging the intestinal barrier, which is mediated by gut microbiota and its metabolites, such as PA. These interactions amplify metabolic dysfunction and inflammatory responses. Moreover, HFD and LDR synergistically contribute to cardiovascular disease, particularly atherosclerosis, by promoting lipid dysregulation and activating the cGAS-STING signaling pathway, which amplifies inflammatory responses and accelerates vascular damage. Further studies have shown that HFD increases radiosensitivity, especially in females, by altering gut microbiota composition and its metabolites, while also exacerbating the inflammatory and metabolic effects of IR exposure. Together, these findings highlight the substantial influence of dietary factors on the biological responses to IR and underscore key mechanisms, including epigenetic regulation, oxidative stress, hormonal disruptions, and gut microbiota interactions, that contribute to IR-induced metabolic and systemic dysfunctions. These insights emphasize the need for targeted interventions, such as dietary modulation or exercise, to mitigate the adverse health effects of IR, particularly in individuals with HFDs, obesity, or cancer survivors undergoing radiation therapy.

### 4.4. A Maternal HFD Modulates the Radiosensitivity of Offspring

#### 4.4.1. Overview

Chronic consumption of HFD is a significant risk factor for one’s own health, leading to obesity and various metabolic syndromes, such as insulin resistance, type 2 diabetes and nonalcoholic fatty liver disease, as well as cancer initiation at multiple organ sites [180,181,182,183]. Moreover, an accumulating body of evidence indicates that maternal consumption of HFD or obesity exerts persistent effects on long-term health of offspring [184]. The gestational and lactational periods are critical for lifelong health because nutrients and other factors needed for offspring growth are transferred from the mother through the placenta to the fetus and through breast milk to the infant, and because rapid growth and development of tissues and organ systems occur during this time. This concept is broadly known as the “developmental origins of health and disease” (DOHaD) [185,186]. Recently, we found that maternal exposure to HFD reduced the lifespan of male offspring following exposure to X-rays, while maternal HFD alone did not (manuscript in submission). Pathological analysis revealed that the lifespan shortening by maternal HFD after X-irradiation was primarily attributed to early deaths associated with depletion of bone marrow cells and thymic lymphoma within 6 months after X-irradiation. To the best of our knowledge, apart from our observation, there are no other studies showing the modifying effects of maternal HFD on the radiosensitivity of offspring. Here we review the possible mechanisms through which maternal HFD influences the radiosensitivity of offspring.

#### 4.4.2. Mechanisms

Epidemiological studies have shown that maternal obesity is associated with an increased risk of childhood overweight and obesity in offspring [186] and exerts persistent effects on the long-term health of offspring, leading to obesity, coronary heart disease, stroke, type 2 diabetes, and asthma [184]. Consistently, male offspring exposed to maternal HFD in our mouse model showed significantly increased body weight gain at least until 6 months of age. Life shortening of male offspring of HFD dams in our mouse model was mainly caused by depletion of bone marrow cells and thymic lymphoma, suggesting that maternal HFD affects the development and homeostasis of the hematopoietic system of the offspring. Similar observations of impaired fetal hematopoiesis in the liver and bone marrow have been reported in mouse and rhesus macaque models of maternal chronic consumption of HFD [187,188]. There are many reports showing that HFD-induced obesity compromises hematopoiesis in the bone marrow and modulates the immune response of the animals affected by obesity [189,190,191,192]. In addition, it was demonstrated that HFD-induced obesity amplifies IR-induced damage to hematopoietic cells in bone marrow as described above [172,175]. These lines of evidence strongly suggest that maternal chronic consumption of HFD dysregulates the hematopoietic system of male offspring, rendering them susceptible to IR. Here, we discuss possible mechanisms by which maternal HFD affects the hematopoietic system of the offspring. These possible mechanisms are not mutually exclusive.

Research on animal models showed that HFD-induced maternal obesity causes their offspring to exhibit perturbations in metabolic and endocrine systems, an increase in adipose tissue, and chronic inflammation [193,194,195,196,197]. The chronic inflammatory status of HFD-induced obesity has been revealed to be associated with alterations in the proliferation and differentiation of hematopoietic stem cells (HSCs) in the bone marrow of affected individuals [191]. Adipose tissue in the bone marrow constitutes a specific microenvironment for hematopoietic cells. Recently, it was revealed that the plasticity of bone marrow adipocyte plays an important role in the regeneration of HSCs after myeloablation by IR [198]. An increase in adipose tissue in the bone marrow of offspring due to maternal HFD may impair the development and homeostasis of hematopoiesis in physiological situations as well as in stressed conditions following X-irradiation [197,199].

It is known that dietary patterns including HFD are associated with the distinct composition of intestinal microbiota [200]. Maternal microbial metabolites and TLR ligands can be transferred across the placenta to the fetus and through lactation to the neonates and affect the development and differentiation of immune cells in the offspring [201,202]. Maternal microbiota is vertically transmitted to the newborn during its passage through the birth canal, forming the foundation of the offspring’s intestinal microbiota [203,204]. It is revealed in a primate model that maternal HFD persistently alters the offspring microbiome [205]. Changes in the intestinal microbiota induced by HFD or obesity have been shown to modulate the regulation of hematopoiesis in the bone marrow [206,207]. Then, changes in the microbiota of both the mother and the offspring may play an important role in the regulation of hematopoiesis and the immune system of the offspring. Several studies using mouse models demonstrated that alterations in the intestinal microbiota composition can impact the radiosensitivity of the host mice [208].

Epigenetic modifications, including alterations in DNA methylation, post-translational histone modifications, and regulation by non-coding RNAs, have been proposed as key causal molecular mechanisms of DOHaD or developmental programming resulting from maternal exposure to various environmental stresses, such as malnutrition and obesity [209]. These epigenetic modifications induced by maternal environmental stresses can occur in oocytes before conception, in the fertilized ovum before implantation, in placental tissues, and in the tissues of offspring during gestational and lactational periods [210]. Notably, alterations in DNA methylation and histone modifications following maternal exposure to an HFD or maternal obesity have been reported in unfertilized oocytes, the placenta, adipose tissue, and the liver of offspring [211,212,213,214,215,216,217,218,219,220].

In our mouse model of chronic maternal exposure to HFD, modulation of radiosensitivity was observed in male offspring but not in females. Sexual dimorphism in DOHaD or developmental programming resulting from maternal exposure to various environmental stresses including obesity and HFD have been reported across a range of organisms, including rodent and human [187,221,222,223]. Changes in DNA methylation and global gene expression patterns following maternal HFD occur more significantly in the placenta of female offspring than in that of male offspring [213,224]. It is proposed that female feto-placental units are more adaptive to maternal HFD exposure than male ones [225]. Table 4 summarizes the possible mechanisms through which maternal HFD influences the radiosensitivity of offspring.

#### 4.4.3. Summary

Our recent observation demonstrated that chronic consumption of an HFD by mother mice renders their male offspring susceptible to bone marrow cell depletion and thymic lymphoma induction following X-TBI. Prenatal and perinatal exposure to environmental stresses is known to have adverse impacts on the lifelong health of offspring and is called DOHaD. Maternal HFD has been shown to have various effects on reproductive tissues, including ovarian follicles and mature oocytes [226]. Notably, just three weeks of HFD consumption is sufficient to increase lipid droplets, which are neutral lipid storage organelles essential for energy metabolism, as well as intracellular triacylglycerol levels in oocytes [227]. During pregnancy and lactation, the mother provides nutrients and other essential factors for offspring growth primarily through the placenta in utero and through breast milk after birth. Since tissues and organs develop rapidly during these periods, maternal HFD is likely to have various impacts on the lifelong health of offspring. Changes in epigenetic processes as well as in the intestinal microbiome have been proposed as the causal molecular mechanisms of DOHaD. In fact, maternal HFD alters the composition of the intestinal microbiome in both the mother and offspring and induces epigenetic changes in preconceptual oocytes of the mother, as well as in the placenta, adipose tissues, and liver of the offspring. It is also reported that maternal HFD leads to perturbations in metabolic and endocrine systems, systemic chronic inflammation, and increase in adipose tissue in offspring. Systemic chronic inflammation may affect proliferation and differentiation of HSCs in the bone marrow. Since adipose tissue in the bone marrow constitutes a specific microenvironment for hematopoietic cells, an increase in adipose tissue may impair the development and homeostasis of hematopoiesis. These are the probable causes by which a maternal HFD renders their offspring susceptible to bone marrow cell depletion and thymic lymphoma induction following exposure to IR in our mouse model. To the best of our knowledge, apart from our observation (manuscript in submission), there are no other studies showing that maternal environmental stress can modify the radiosensitivity of offspring. The current radiation protection system does not consider individual differences in radiation sensitivity. In particular, there is strong public interest in children’s radiation sensitivity, highlighting the need for protective measures that take these differences into account. Further research is necessary to better understand how maternal environmental stress or health influences the biological effects of IR on offspring.

### 4.5. Overall Summary and Conclusions

IR causes metabolic dysfunctions such as obesity, insulin resistance, and dysregulated lipid metabolism. In animal models, sublethal WBI in rhesus macaques and low-dose-rate IR exposure in mice resulted in metabolic disturbances, including diabetes and weight gain, with effects on insulin signaling, inflammation, and adipocyte function. Studies in pigs showed systemic metabolic issues linked to cardiovascular dysfunction. These findings highlight the need for further research into the complex mechanisms underlying IR-induced metabolic diseases.

IR has shown therapeutic potential in addressing metabolic disorders caused by HFDs. In models, localized IR helped reduce atherosclerosis and kidney damage induced by diabetes. LDR demonstrated renoprotective effects and anti-inflammatory properties, improving lipid metabolism and glucose regulation. These findings suggest that IR could be used in novel interventions to address HFD-related metabolic conditions.

The combination of HFD and IR exacerbates cardiovascular and metabolic diseases. HFDs alone contribute to lipid metabolism disturbances and atherosclerosis, while IR worsens these conditions by increasing oxidative stress, lipid deposition, and inflammation. Studies indicate that dietary fats influence the severity of IR’s impact, and epigenetic changes related to HFDs further sensitize tissues to IR. Additionally, obesity and HFDs exacerbate radiation-induced damage to hematopoietic cells and organs, increasing the risk of chronic diseases. Research highlights the need for interventions, such as dietary adjustments and exercise, to mitigate the harmful effects of IR, particularly in those with obesity or metabolic disorders.

Emerging evidence also shows that maternal HFD significantly alters the radiosensitivity of offspring. Recent studies in mouse models suggested that maternal HFD shortens the lifespan of male offspring after X-ray exposure, which primarily contributed to early death associated with bone marrow cell depletion and thymic lymphoma. Radiation-induced bone marrow suppression seems to be mediated by dysregulation of the hematopoietic system, which maternal HFD drives via mechanisms including chronic inflammation, obesity-associated bone marrow adiposity, microbiome alterations, and epigenetic changes. This radiosensitivity appears to be mediated by dysregulation of the hematopoietic system, influenced by maternal HFD through mechanisms such as chronic inflammation, alterations in bone marrow adiposity, microbiome shifts, and epigenetic modifications. The DOHaD framework provides a critical lens for understanding how maternal environmental factors, including diet, affect offspring health. Maternal HFD-induced changes in intestinal microbiota and epigenetic landscapes can significantly impact hematopoiesis and immune function in offspring, thereby modulating their radiosensitivity. Of particular note, sexual dimorphism has been demonstrated, with males exhibiting more IR-induced damage than females.

In summary, the interplay between IR and HFDs has profound health implications, with IR both exacerbating and alleviating metabolic dysfunctions. The effect of maternal HFD on radiosensitivity in their offspring adds another layer of complexity to the need for systematic approaches to radiosensitizing dietary components and physical activity, as well as personalized measures to mitigate radiation hazard during cancer treatment or to face it in the context of working or living in radiation-polluted environments. Future research should focus on unraveling the intricate mechanisms by which maternal health and environmental factors influence IR responses, ultimately guiding the development of targeted interventions to protect vulnerable populations.

## 5. Clinical and Public Health Implications

The integration of dietary interventions to mitigate the adverse effects of IR garnered substantial attention in recent research. This field bridges nutrition and radiation science, offering promising pathways to enhance radiation protection and therapeutic efficacy.

IR serves as a critical tool in healthcare for diagnostics and treatment. However, its use comes with inherent risks, particularly at high doses, which can cause DNA damage, oxidative stress, genotoxicity, inflammation, and carcinogenesis. The risks are evident in both therapeutic contexts, such as radiotherapy, and accidental exposures. Similarly, HFDs exacerbate these risks, as they have been linked to increased oxidative stress, DNA damage, and systemic inflammation, further contributing to carcinogenesis. A balanced diet thus emerges as a crucial factor in mitigating these adverse effects. This review examines the interplay between HFDs and IR exposure, highlighting the importance of dietary interventions in reducing associated health risks.

Experimental studies in animal models underscore the potential of dietary interventions in alleviating deterministic effects of IR and lowering carcinogenesis risks in specific tumor types. Dietary components such as bioactive compounds, antioxidants, and micronutrients demonstrated dual benefits, protecting healthy tissues while enhancing the therapeutic efficacy of radiation treatments. For instance, antioxidants mitigate oxidative stress and promote DNA repair, offering a radioprotective advantage. Emerging evidence suggests that targeted dietary strategies, customized for individuals exposed to radiation, could complement current radiation protection protocols and improve health outcomes [228].

The concept of “proactive radiation protection” highlights the importance of applying transdisciplinary research to prevent radiation-induced damage and reduce health risks. This proactive approach leverages medical interventions to maximize radiation protection potential. Successful cancer treatment, for example, often relies on multimodal strategies that integrate proactive radiation protection with other therapeutic modalities [229]. Although radiotherapy as cornerstone of nonsurgical cancer management achieved significant advancements in both methodology and biological understanding, its major limitation is the potential for secondary malignancies induced by radiation exposure. Addressing this drawback requires a comprehensive, integrative effort. Multimodal approaches combine radiation therapy with pharmacological agents such as tissue-specific radiosensitizers and radioprotectors. Additionally, novel methods such as gene therapy, induction of hormesis, and adaptive responses are being explored. Lifestyle interventions, including dietary modifications and the use of dietary restriction mimetic drugs, psychiatric care, and public health initiatives, further broaden the spectrum of therapeutic strategies. Among the many modalities, dietary interventions stand out for their feasibility and broad acceptance. As an adjuvant therapy, dietary strategies have the potential to complement radiation protection measures, reduce side effects, and enhance therapeutic outcomes. For example, bioactive compounds such as polyphenols, vitamins, and trace elements have shown promise in experimental and preclinical studies [228,230].

The convergence of nutrition and radiation science offers innovative opportunities to improve radiation protection and therapy. By leveraging the radioprotective properties of dietary interventions, it is possible to mitigate IR-induced health risks effectively. Future research should prioritize developing evidence-based dietary guidelines tailored to varying radiation exposure levels, investigating the synergistic effects of dietary and pharmacological interventions, and conducting robust clinical trials to establish efficacy and optimize dietary regimens. Of note, the translation of dietary interventions into clinical settings remains a challenge due to individual variability and the complexity of interactions between diet and radiation. Nevertheless, these advancements hold transformative potential for improving patient care, occupational safety, and public health outcomes.

## 6. Conclusions

This review highlights the complex interplay between HFDs and IR, revealing their roles in aggravating and alleviating metabolic dysfunctions. IR exposure is linked to metabolic disturbances, such as obesity, insulin resistance, and dysregulated lipid metabolism, as observed in animal models. Paradoxically, IR may also alleviate certain HFD-induced metabolic disorders through its renoprotective and anti-inflammatory effects, improving lipid metabolism and glucose regulation. However, the combination of HFD and IR often exacerbates cardiovascular and metabolic diseases by increasing oxidative stress, lipid deposition, and inflammation. HFDs can heighten IR sensitivity through epigenetic modifications and exacerbate IR-induced damage to hematopoietic cells and organs, thereby increasing the risk of chronic diseases. Maternal HFD further modulates offspring radiosensitivity, affecting hematopoietic development through chronic inflammation, gut microbiota alterations, and epigenetic modifications, with male offspring being particularly vulnerable.

Together, these findings underscore the significant health implications of IR in the context of HFDs and highlight the need for further investigation into the underlying mechanisms and potential targeted interventions. A deeper understanding of these interactions will not only enhance radiation threat assessments, but also inform public health strategies and improve therapeutic outcomes, especially for vulnerable populations such as children and individuals with metabolic disorders. Integrating dietary modulations with proactive radiation protection strategies offers a promising avenue for mitigating the compounded health risks associated with IR and HFDs, providing reassurance and confidence in the potential for effective solutions.

## Figures and Tables

**Table 1:** Mechanisms underlying IR-induced obesity and metabolic dysregulation.

Organ/System	Mechanisms	Details	References
Metabolic system	Insulin resistance and lipid dysregulation	IR disrupted insulin signaling by reducing IRS-1 phosphorylation in subcutaneous adipose tissue and increasing basal lipolysis, leading to adipocyte hypertrophy, macrophage infiltration, and reduced adiponectin levels	[148,151]
	Mitochondrial dysfunction	IR reduced mtDNA copy numbers and increased mtDNA mutations, impairing energy balance, promoting fat storage, and disrupting systemic lipid metabolism	[150,151]
	Hormonal dysregulation	IR reduced IGF-1 signaling and downregulates CPT1 and IRS2, critical for lipid metabolism and insulin sensitivity, leading to obesity, insulin resistance, and systemic metabolic syndrome	[152]
Adipose tissue	Hepatic inflammation mitigation	Enlarged adipocytes (hypertrophy), increased lipolysis, and macrophage-driven inflammation disrupt glycemic control	[148]
	Absence of fibrosis	IR-induced metabolic dysfunction occurred without evidence of adipose tissue fibrosis, highlighting alternative pathways	[148]
Liver	Lipid accumulation	IR increased liver lipid content and serum leptin levels, reflecting systemic lipid dysregulation independent of food intake	[150,151]
Cardiovascular system	IGF-1 signaling impairment	Reduced cardiac IGF-1 signaling contributed to metabolic syndrome and cardiovascular dysfunction, paralleling HFD outcomes	[152]
	TGF-β1-mediated fibrosis	Elevated TGF-β1 levels exacerbated tissue damage and fibrosis, impairing organ function and promoting cardiovascular abnormalities	[152]
Systemic effects	Chronic inflammation and adiposity	IR-induced inflammation and systemic lipid dysregulation contributed to adiposity and metabolic syndrome, resembling HFD outcomes	[147,151]
	Dose-dependent weight gain	Chronic low-dose-rate IR exposure caused weight gain in a dose-dependent manner, alongside increased adiposity and disrupted lipid profiles	[149,150]
	Metabolic syndrome	IR exposure lead to systemic metabolic dysfunction, including altered lipid profiles, reduced IGF-1 levels, and increased leptin, contributing to obesity and related disorders	[147,151]
Endocrine system	Disrupted hormonal	IR reduced circulating IGF-1 and leptin signaling dysregulation reflecting systemic endocrine dysfunction, contributing to obesity and metabolic abnormalities	[151,152]

**Table 2:** The mechanisms of IR-induced alleviation of HFD-induced metabolic effects across organ systems.

Organ/System	Mechanisms	Details	References
Cardiovascular system	Reduction in atherosclerosis	High-dose localized irradiation reduced atherosclerotic lesions in cholesterol-fed rabbits by suppressing intimal hyperplasia	[153,154]
	Inhibition of atherosclerosis	LDR reduced neutrophil extracellular traps, decreasing endothelial plaque formation in atherosclerosis models by modulating immune pathways and inflammation	[163]
	Reduction in inflammation	LDR reduced pro-inflammatory cytokines while increasing anti-inflammatory markers, helping suppress cardiovascular inflammation	[159]
Hepatic system	Hepatic inflammation mitigation	FWBGI reduced liver inflammation, increased anti-inflammatory cytokines, and modulated gene expression to improve insulin sensitivity and energy balance	[159,160]
Endocrine system	Enhanced glucose metabolism and insulin sensitivity	FWBGI increased GLP-1 expression, upregulated GLP-1 receptors in the liver and brain, and activated mitochondrial uncoupling proteins, improving insulin sensitivity and energy balance	[161]
Systemic metabolic effects	Systemic metabolic benefits	LDR improved dyslipidemia, insulin resistance, and kidney pathology by enhancing protein kinase B activation and Nrf2-mediated antioxidant responses	[157,158]
	Anti-inflammatory effects	FWBGI suppressed pro-inflammatory cytokines while promoting anti-inflammatory balance	[159]
Lipid metabolism	Modulation of lipid metabolism	IR altered lipid remodeling processes, with HFD partially mitigating IR-induced skin injuries; Adipocytes facilitated tissue repair via fatty acid-binding protein 4 and palmitic acid pathways	[162]

**Table 3:** The mechanisms underlying enhancement of IR-induced detrimental effects by HFDs across organ systems.

Organ/System	Mechanisms	Details	References
Cardiovascular system	Lipid dysregulation and atherosclerosis	HFD elevated LDL, reduced HDL, and induced lipid deposition in arterial walls; when combined with IR, leading to oxidative stress, inflammation, smooth muscle cell proliferation, and accelerating atherosclerotic lesions in coronary arteries	[164,165,168,169,178,179]
	Oxidative stress and vascular inflammation	IR and HFD synergistically enhanced oxidative stress and vascular inflammation, promoting arterial injury and plaque formation	[166,167,168]
Metabolic system	Insulin resistance and glucose dysregulation	HFD disrupted glucose metabolism and insulin signaling in skeletal muscle and adipose progenitor cells; IR exacerbated these metabolic dysfunctions through persistent intracellular signaling disruptions	[173,176]
	Epigenetic reprogramming	HFD altered methylation of gene promoters and disrupts microRNA, leading to radiosensitization and impaired adaptive responses to IR	[171]
	Gut microbiota dysregulation	HFD reduced microbial diversity, increased inflammation, and exacerbated systemic IR-induced damage; synergistic effects worsened insulin resistance and intestinal barrier dysfunction, especially in female mice	[12,176,177]
Liver	Oxidative stress, inflammation, and fibrosis	HFD amplified IR-induced liver damage, leading to heightened inflammation, oxidative stress, fibrosis, and increased cancer risk	[174]
Hematopoietic system	Suppression of hematopoietic stem and progenitor cells	LDR improved dyslipidemia, insulin resistance, and kidney pathology by enhancing protein kinase B activation and Nrf2-mediated antioxidant responses	[175]
	Increase in radiosensitivity and genotoxicity	HFD increased radiosensitivity, as evidenced by reduced survival following WBI and persistent presence of micronucleated erythrocytes in bone marrow	[172]
Intestinal system	Intestinal barrier dysfunction	HFD combined with IR damaged the intestinal barrier, reduced stem cell regeneration, and altered gut microbiota, worsening systemic inflammation and recovery; effects were more pronounced in female mice	[13,177]
Systemic effects	Chronic inflammation	HFD synergized with IR induced chronic inflammation via cGAS-STING signaling, leading to systemic oxidative stress, vascular stiffness, and enhanced inflammatory responses	[178]
	Decrease in survival post-IR	HFD diminished the adaptive response to IR, reducing survival rates and increasing the LD50.	[172]

**Table 4:** Possible mechanisms through which maternal HFD influences the radiosensitivity of offspring.

Organ/System	Mechanisms	Details	References
Hematopoietic tissues	HFD-induced obesity can affect the hematopoietic system not only in affected individuals themselves but also in their offspring	These papers demonstrate in the mouse models that HFD-induced obesity compromises hematopoiesis in the bone marrow, modulates immune responses, and sensitizes affected individuals to environmental stresses such as IR	[172,175,189,190,191,192]
		These papers demonstrate in the moue and rhesus macaque models that maternal HFD compromises the proliferation and differentiation of HSPCs in the fetal liver and bone marrow	[187,188]
Endocrine and metabolic systems, adipose tissues, hematopoietic tissues	Maternal HFD induces perturbations in metabolic and endocrine systems, chronic inflammation, increased adipose tissue, and ultimately impacts the hematopoietic system of offspring	Using mouse or Japanese macaque model, these papers show that maternal HFD induces perturbations in metabolic and endocrine systems regulating lipid and glucose metabolism, leads to an increase in adipose tissues, and triggers inflammatory responses in the liver and adipose tissues	[193,194,195]
		This paper links chronic inflammation to altered HSC proliferation and differentiation in HFD-induced obese mice themselves	[191]
		These papers show that an increase in adipose tissue in the bone marrow of HFD-induced obese mice impairs the development and homeostasis of hematopoiesis, as adipose tissue in the bone marrow constitutes a specific microenvironment for hematopoietic cells	[198,199]
Intestinal microbiota and hematopoietic and immune systems	Maternal HFD modulates the composition of intestinal microbiota in the mother herself as well as in their offspring; intestinal microbiota plays an important role in the development of the immune system	This review article summarizes the modulating effects of a Western-style diet on the composition of the human intestinal microbiota	[200]
		These papers show that the maternal microbial metabolites can be transferred across the placental barrier to the fetus and through lactation to the neonates and shapes the immune system of the offspring	[201,202]
		This paper shows that a maternal HFD persistently alters the offspring microbiome in a primate model (Japanese macaque)	[205]
		These papers demonstrate in mice and humans that the colonization of offspring’s microbiota is initiated by the vertical transmission of microbiota during their passage through the birth canal	[203,204]
		These papers show that HFD- or obesity-induced changes in intestinal microbiota affect the regulation of hematopoiesis in bone marrow	[206,207]
		This review summarizes studies in mouse models demonstrating that changes in the gut microbiota can influence the radiosusceptibility of the host mice	[208]
Oocytes, plasence, adipose tissues, and liver	Maternal HFD induces the epigenetic changes in oocyets, placenta and adipose tissues, and liver of offspring	These papers demonstrate the changes in DNA methylation in oocytes of HFD-induced obese mice	[211,212]
		This paper demonstrates that maternal HFD influences placental methylation patterns only in female offspring of mice	[213]
		These papers demonstrate alterations in DNA methylation and histone modifications in adipose tissues following maternal HFD consumption or obesity in mice and rats	[214,215,216]
		These papers demonstrate alterations in DNA methylation and histone modifications in liver following exposure to maternal HFD in mice, rats and Japanese macaques	[217,218,219,220]
		These papers demonstrate sexual dimorphism in epigenetic changes in the placenta following maternal HFD in mice	[213,224]

## Data Availability

Not applicable.
